# Dissecting the mycobacterial cell envelope and defining the composition of the native mycomembrane

**DOI:** 10.1038/s41598-017-12718-4

**Published:** 2017-10-09

**Authors:** Laura Chiaradia, Cyril Lefebvre, Julien Parra, Julien Marcoux, Odile Burlet-Schiltz, Gilles Etienne, Maryelle Tropis, Mamadou Daffé

**Affiliations:** 1 Institut de Pharmacologie et de Biologie Structurale, Université de Toulouse, CNRS, UPS, Toulouse, France; 2 Université de Toulouse, UPS, IPBS, 31000 Toulouse, France

## Abstract

The mycobacterial envelope is unique, containing the so-called mycomembrane (MM) composed of very-long chain fatty acids, mycolic acids (MA). Presently, the molecular composition of the MM remains unproven, due to the diversity of methods used for determining its composition. The plasma membranes (PM) and the native MM-containing cell walls (MMCW) of two rapid-growing mycobacterial species, *Mycobacterium aurum* and *M. smegmatis*, were isolated from their cell lysates by differential ultracentrifugation. Transmission electron microscopy and biochemical analyses demonstrated that the two membranes were virtually pure. Bottom-up quantitative proteomics study indicated a different distribution of more than 2,100 proteins between the PM and MMCW. Among these, the mannosyltransferase PimB, galactofuranosyltransferase GlfT2, Cytochrome p450 and ABC transporter YjfF, were most abundant in the PM, which also contain lipoglycans, phospholipids, including phosphatidylinositol mannosides, and only a tiny amount of other glycolipids. Antigen85 complex proteins, porins and the putative transporters MCE protein family were mostly found in MMCW fraction that contains MA esterifying arabinogalactan, constituting the inner leaflet of MM. Glycolipids, phospholipids and lipoglycans, together with proteins, presumably composed the outer leaflet of the MM, a lipid composition that differs from that deduced from the widely used extraction method of mycobacterial cells with dioctylsulfosuccinate sodium.

## Introduction

Mycobacteria are probably the most successful microorganisms to parasite animals and humans. Among the 187 valid species described to date in the genus *Mycobacterium*, only three are strict pathogens for human: *Mycobacterium tuberculosis* (*Mtu*), *M. leprae* and *M. lepromatosis* ^1^. Tuberculosis still represents a major public health problem worldwide, remaining one of the world leading causes of death from an infectious agent, about one third of the world population being infected by the Koch bacillus and susceptible to develop the disease. In addition, two-thirds of mycobacteria species are opportunistic pathogens for human, and with large enough inoculum, all mycobacteria produce granulomatous lesions in experimental animals^2^.

The cell envelope is critical for the mycobacterial physiology, primarily because many crucial processes are located in this compartment. These include the protection of the bacterial cell from hostile environments, mechanical resistance of the cells, transport of solutes and proteins, adhesion to receptors. The hallmark of mycobacteria is their unique abundance in lipid, constituting up to 40% of the dry weight of the tubercle bacillus^3,4^. The mycobacterial cell wall contains up to 60% of lipids, as compared with some 20% for the lipid-rich cell walls of Gram-negative microorganisms^4^. These lipids include the exceptionally-long chain fatty acids (mycolic acids, MA) covalently linked to the cell wall polysaccharide arabinogalacatan (AG) and whose esterifying trehalose, as well as the numerous classes of exotic compounds typifying the *Mycobacterium* genus. To these lipids have been attributed many of the biological properties of mycobacteria^3,4^. These include the very high resistance of the majority of mycobacterial species to most of the broad-spectrum antibiotics, except for instance streptomycin and rifamycins^2^and their recognized impermeability to nutrients, up to 100- to 1,000-fold less permeable than the most resistant Gram-negative bacteria *Escherichia coli* and *Pseudomonas aeruginosa* ^5^.

**Figure 1:**
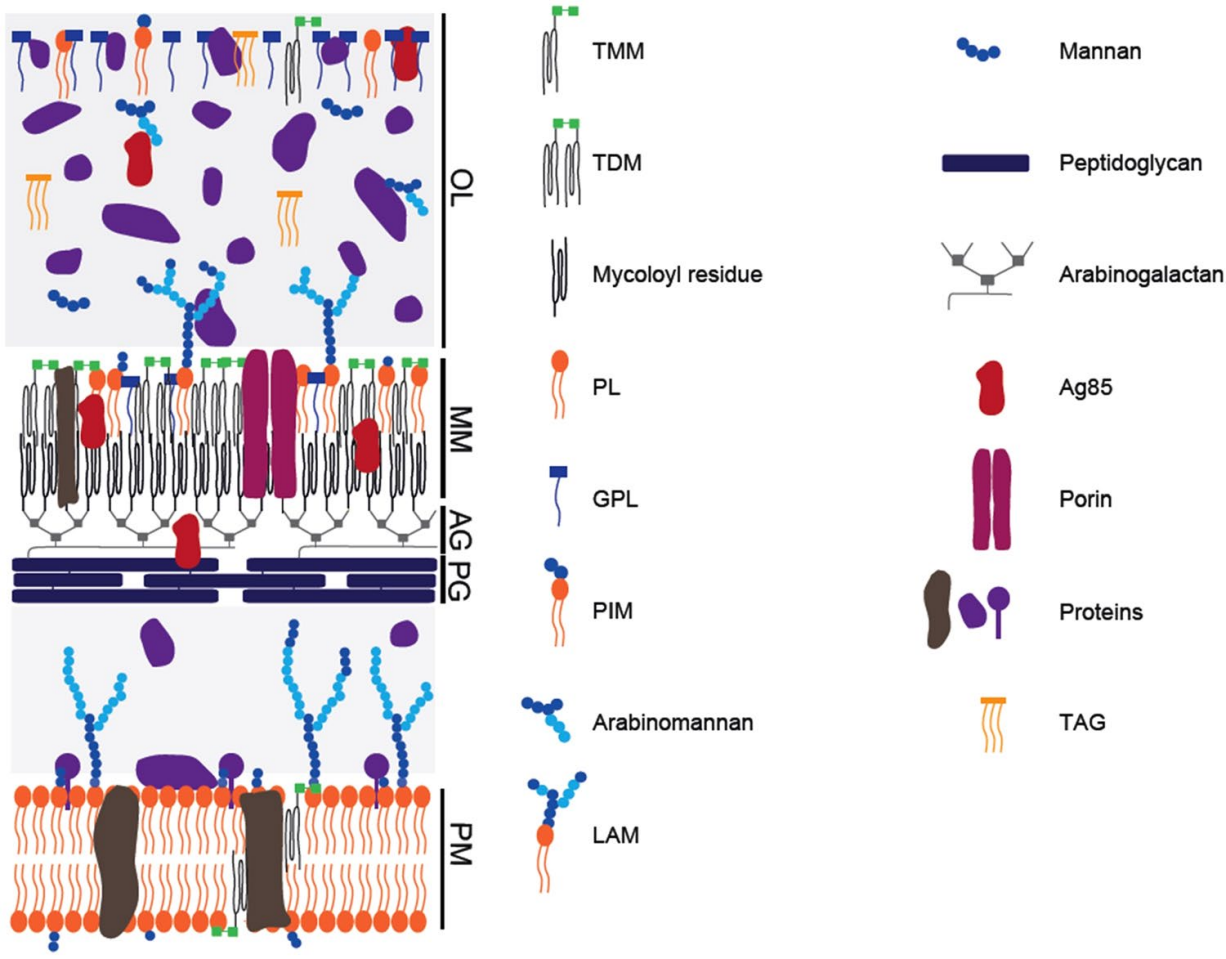
A tentative model of the arrangement of the cell envelope of *M. smegmatis*. (adapted from^11^). The outermost layer (OL) is primarily constituted of proteins, with small amounts of carbohydrate and only a tiny amount of lipids. The cell wall is a giant tripartite complex composed of the outer membrane, the so-called mycomembrane (MM), arabinogalactan (AG) and peptidoglycan (PG). The inner leaflet of the MM is made of very long-chain fatty acids (mycolic acids) esterifying AG, which in turn is covalently attached to PG. The outer leaflet of the MM is presumably composed of lipids extractable with organic solvents, which include phospholipids, trehalose mycolates, glycopeptidolipids, and lipoglycans. A periplasmic space separates the cell wall from the conventional lipid bilayer PM of phospholipids and proteins whose thickness is surprisingly similar to that of MM, around 7–8 nm, despite the presence of the very long chain mycolic acids. The scales of the various cell envelope compartments are based on the data from CEMOVIS^6^, except that of the OL, which is adapted from cryo-microscopy. For clarity, molecules are not drawn on scale. TMM : trehalose monomycolates; TDM: trehalose dimycolates; GPL: glycopeptidolipids; PL: phospholipids; PIM: phosphatidyl-myo-inositol-mannosides ; LAM: lipoarabinomannans; TAG: triacylglycerols; Ag85: antigen 85.

Despite its clearly established importance, little is known on the composition and arrangement of mycobacterial cell envelope constituents, especially when compared to the vast knowledge acquired on the cell envelope of Gram-negative bacteria. For instance, it is only recently that the presence of an outer membrane, also called mycomembrane (MM), has been demonstrated^6–8^ by cryo-electron microscopy of vitreous sections (CEMOVIS), a very unusual feature for bacteria that belong to the *Actinobacteria* phylum of Gram-positive (monoderm) bacteria. Different models have been proposed for the mycobacterial cell envelope^3,6–10^, which are greatly dependent on the determination of its exact composition. The most recent model^11^ schematically divides the mycobacterial cell envelope in three entities (Fig. 1): an outermost layer (OL), also called capsule in the case of pathogenic species^3,12^, a cell wall (CW) and a conventional plasma membrane (PM). The capsule of mycobacterial pathogens such as *Mtu* is mainly composed of glucan and proteins, with only a tiny amount of lipids whereas the OL of non-pathogens is primarily constituted of proteins^13–15^. The CW is a giant tripartite complex composed of the MM, AG and peptidoglycan (PG), also known as the mAGP complex^3^. The MM exhibits a non-conventional bilayer organization in which the inner leaflet is made of very long-chain MA linked to AG, which in turn is covalently attached to PG. The outer leaflet of the MM is presumably composed of free - *i.e*. non-covalently bound to the cell – lipids^3,9,11^. A periplasmic space separates the CW from the conventional lipid bilayer PM^6^. Importantly, others and we have demonstrated that the MM thickness is around 7–8 nm^6,8^ despite the presence of the very long chain of MA (up to C90), which raises important questions about the exact native conformation of MA. This outer membrane is also supposed to contain porins for the uptake of small hydrophilic molecules – porins characterized only in the case of *M. smegmatis* (*Msm*)^16^ and membrane machineries involved in the secretion of virulence factors such as the well described ESAT-6 and Cfp10 proteins^17–19^, whose mechanism of translocation across the MM remains largely unknown. In contrast to the OL and MM, the PM has been well studied and proved to be similar of those to other microorganisms^3^. Therefore, determining the composition, localization and supramolecular organization of outermost layer and MM constituents would represent an important and highly significant advance in our knowledge of the mycobacteria physiology and pathogenicity.

In a previous work, we have compared different methods for lysis and fractionating mycobacteria cells^20^, determining the purity of subcellular fractions by using specific PM and CW markers. However, it remained necessary to obtain more purified CW and PM fractions to gain insight into the molecular composition of the cell envelope, which in turn determines the final arrangement of the constituents and its architecture. The only pure MM-containing CW (MMCW), *i.e*. the envelope devoid of the PM, isolated and characterized so far is that from a species belonging to the early branching *Corynebacterium* genus in the phylogenetic tree of *Corynebacteriales*, using separation of membrane fractions by isopycnic sucrose gradient centrifugation^21^. As a proof of the principle, we combined various methods to isolate, for the first time, the MMCW of two mycobacterial model species, namely *M. aurum* (*Mau*) and *Msm*, virtually free from significant contamination by the PM and identify the lipids constitutive of the mycomembrane.

## Results

### Fractionation of mycobacterial cell envelopes on a sucrose gradient and identification of the fractions

The MMCW of *C. glutamicum* (*Cgl*) have been separated from the PM by isopycnic sucrose gradient centrifugation of the lysate obtained after breaking the cells^21^. We adapted this technique to the fractionation of the cell envelopes of *Mau* and *Msm*. Preliminary experiments showed that the growth phase had little effect, if any, on the quality of the membrane fractions. Accordingly, the purification process was performed with mycobacterial cells harvested in the logarithmic phase of growth, ensuring a homogeneous physiological state of the cells.

**Figure 2:**
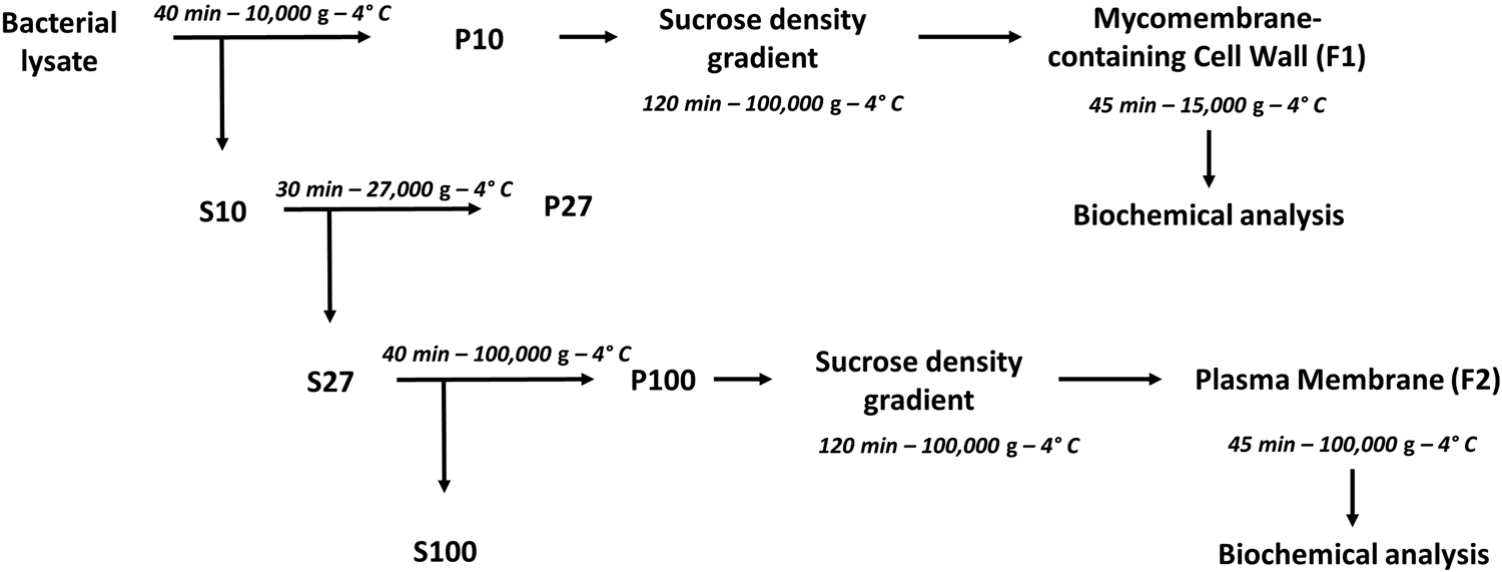
A schematic representation of the fractionation of the mycobacterial lysate for isolating the MMCW and the PM. After mechanical breaking, the bacterial lysate was centrifuged to yield crude cell wall fraction (pellet P10), which was then layered on a sucrose step gradient and centrifuged to recover the cell walls. The S10 supernatant was centrifuged and the S27 supernatant was used to collect the crude PM fraction (pellet P100), which was then layered on a sucrose step gradient.

**Figure 3:**
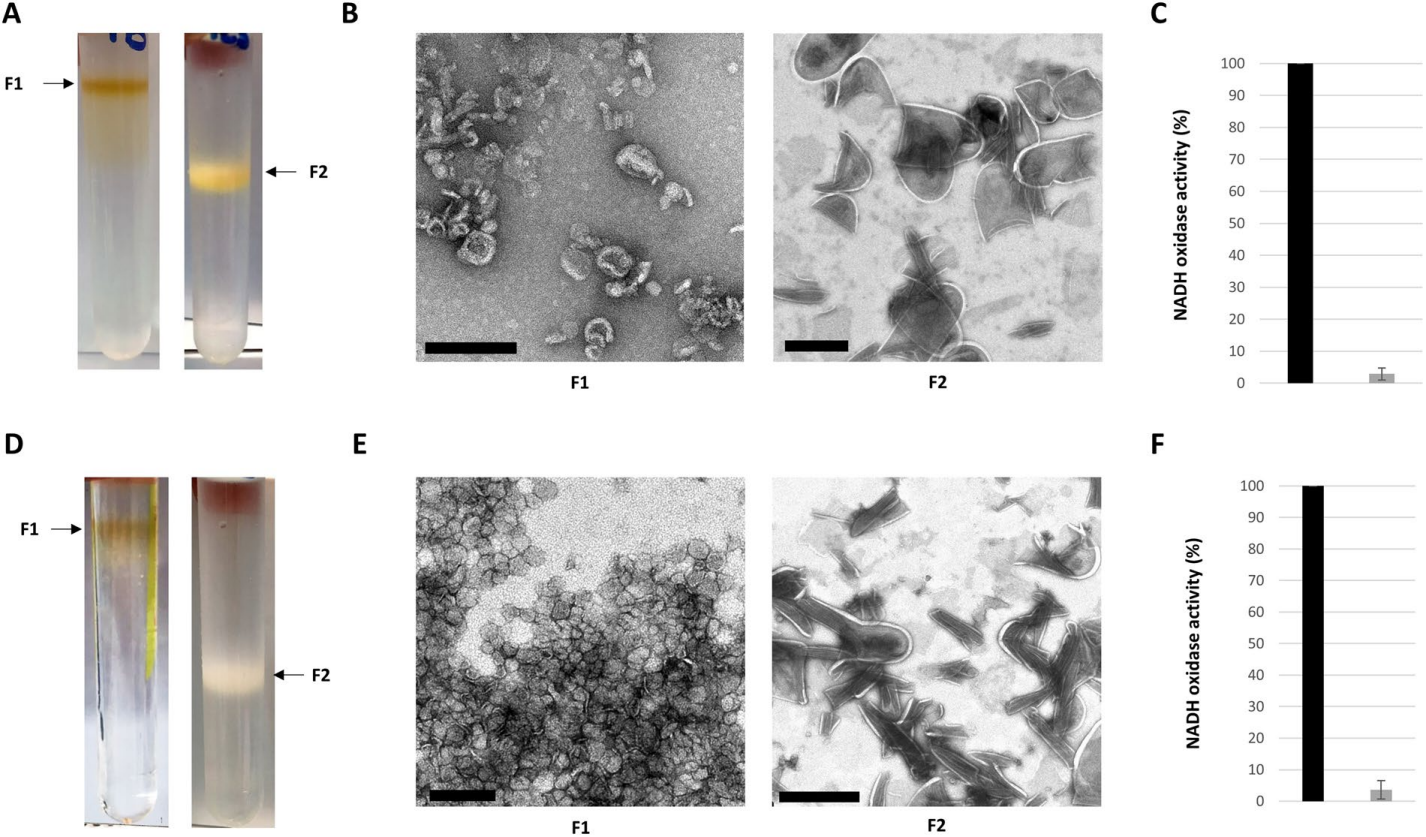
Isolation and NADH oxidase activity of mycobacterial membranes. Visualization of the F1 and F2 of *Mau* (**A**) and *Msm* (**D**). Negative staining of the F1 and F2 fractions of *Mau* (**B**) and *Msm* (**E**); bars represent 200 nm for F1 and 1 µm for F2. NADH oxidase activity of F1 (black symbols) and F2 (grey symbols) of *Mau* (**C**) and *Msm* (**F**).

Disruption of the mycobacterial cells in a French press remains the best lysis method for obtaining significant amounts of cell envelope fractions from these bacteria^20^. The membrane fractions of *Mau* were recovered from the cell lysate by differential ultracentrifugation (Fig. 2). The 10,000 *g* pellet (P10) presumably contained the crude MMCW whereas the PM was expected into the 100,000 *g* pellet (P100). Both fractions were then purified twice by identical sucrose step gradients. More than forty different gradients were tested to finally achieve the best separation of the membrane fractions of *Mau* using a 10% (w/w) to 60% (w/w) sucrose gradient. The gradient consisted of 10% [1 Vol] – 36% [3 Vol] – 40% [3 Vol] – 42% [2 Vol] – 50% [1 Vol] – 60% [1 Vol]. Following centrifugation at 100,000 *g*, two major homogeneous fractions were recovered: a brownish low-density fraction *Mau* F1 (density [*d*] between 1.081 and 1.127 g.cm^−3^), isolated from the P100 pellet, and a light-yellow high-density fraction *Mau* F2 (*d* between 1.167 and 1.176 g.cm^−3^), isolated from P10 (Fig. 3A). In both cases, a white band was observed at the top of the sucrose gradient. Electron microscopy pictures obtained after negative staining with uranyl acetate indicated that F1 and F2 fractions correspond to distinct membrane components of the cell envelope. They contained almost exclusively small liposomes-like particles for *Mau* F1 and very large unclosed fragments for *Mau* F2 (Fig. 3B). Consistently, the activity of NADH oxidase, a respiratory chain enzyme that specifically marks the PM^20,22^, was almost absent from the *Mau* F2, representing less than 3% of the activity measured in fraction *Mau* F1 (Fig. 3C). Taken together, these results suggested that the *Mau* F2 fraction corresponded to the MMCW whereas the *Mau* F1 fraction was assignable to the PM, and that the former fraction was virtually free from significant contamination by the latter one.

Based on their surface-exposed lipid composition^13^, the cell envelope of *Msm* was expected to be more complex than that of *Mau*. Not surprisingly, the sucrose step gradient optimized for *Mau* (10% [1 Vol] – 36% [3 Vol] – 40% [3 Vol] – 42% [2 Vol] – 50% [1 Vol] – 60% [1 Vol]) was poorly effective to separate the two membrane fractions of *Msm*. Several attempts were made to optimize the fractionation protocol, with little success. These included *i)* the use of degradative enzymes such as α-amylase, lysozyme, β-galactosidase, or α-mannosidase to degrade various cell wall constituents, ii) the treatment of grown mycobacteria with Tween 80 prior to their lysis to remove the outermost layer, or the treatment of the crude CW pellet with either urea or NaCl to remove non-specific interacting compounds. Finally, it is the use of an amended 10% (w/w) to 60% (w/w) sucrose gradient, consisting of 10% [1 Vol] – 20% [1 Vol] – 30% [3 Vol] – 36% [3 Vol] – 40% [1 Vol] – 50% [1 Vol] – 60% [1 Vol], that gave, upon centrifugation at 100,000 *g*, two homogeneous fractions of *Msm*: a brownish low-density fraction *Msm* F1 (*d* between 1.081 and 1.127 g.cm^−3^), isolated from the pellet P100, and a white high-density fraction *Msm* F2 isolated from P10 (*d* between 1.127 and 1.167 g.cm^−3^) (Fig. 3D). Again, a white band was observed at the top of the sucrose gradients. *Msm* low- and high-density fractions exhibited appearances in electron microscopy similar to their *Mau* counterparts: small liposomes-like particles for *Msm* F1 and very large unclosed fragments for *Msm* F2 (Fig. 3E). Similarly, *Msm* F2 fractions contained very little NADH oxidase activity (3.6% of that measured in *Msm* F1, Fig. 3F). Therefore, as for *Mau*, the above results suggested that the *Msm* F2 and F1 fractions corresponded, respectively, to the MMCW and PM, and that fraction *Msm* F2 was virtually free from significant contamination by PM.

### Biochemical characterization of membrane fractions

**Figure 4:**
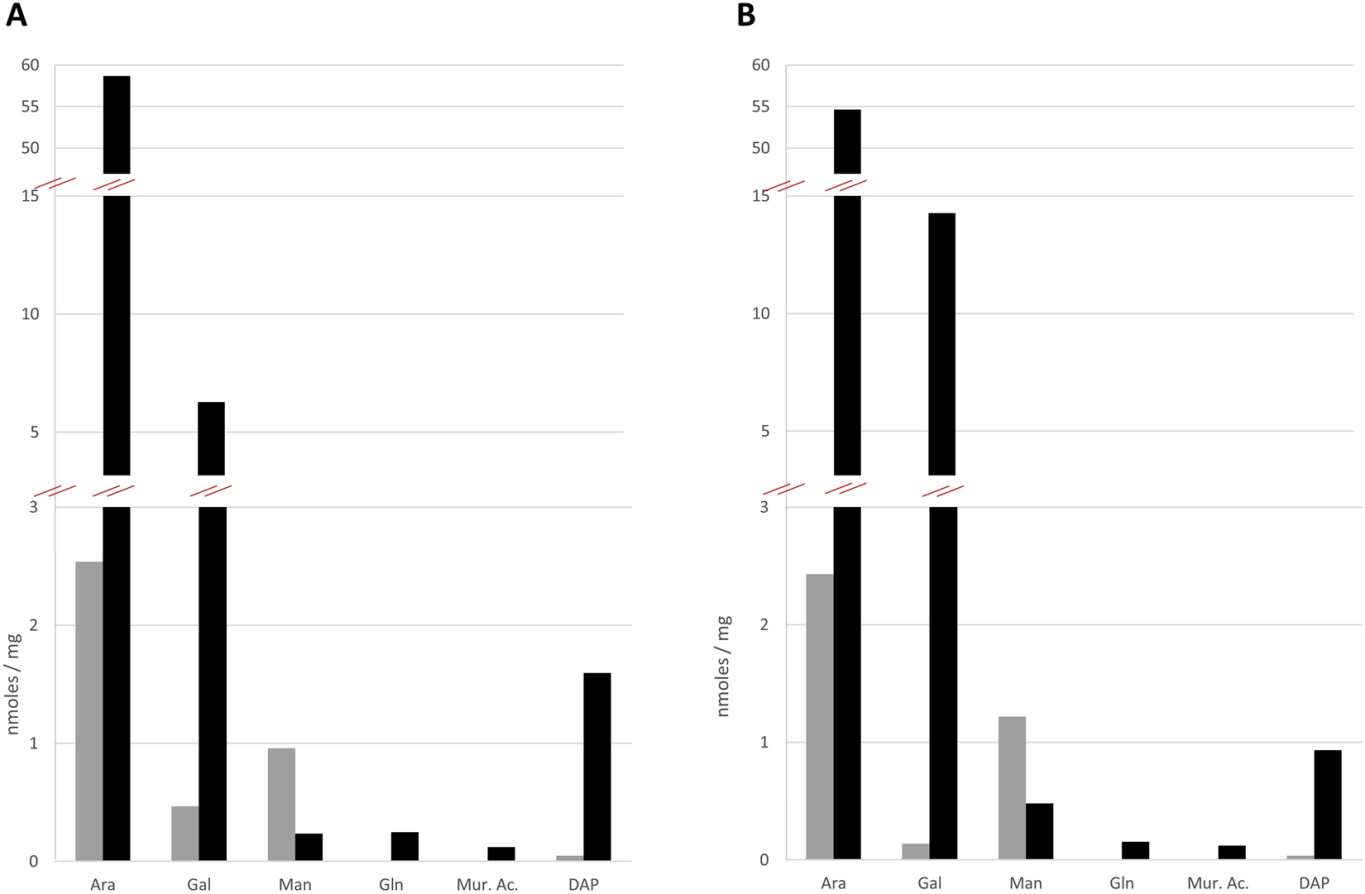
Biochemical characterization of mycobacterial membrane markers. Purified F1 and F2 of *Mau* (**A**) and *Msm* (**B**) were hydrolyzed, derivatized and analyzed by gas chromatography-mass spectrometry, and their contents (nmoles per mg of dried membranes) in galactose (Gal), arabinose (Ara), mannose (Man), glucosamine (Gln), muramic acid (Mur. Ac.) and diaminopimelic acid (DAP) were determined. F1 (grey symbols) and F2 (black symbols).

Although determination of the NADH oxidase activity suggested that the high-density fractions F2 and low-density fractions F1 corresponded to the MMCW and the PM, respectively, the nature of the isolated bands was established by biochemical analyses, *i.e*. determination of the amounts of arabinose and galactose, as AG markers, and glucosamine, muramic acid and diaminopimelic acid (DAP), as PG markers. Firstly, the F1 and F2 dry pellets were subjected to trifluoroacetic acid hydrolysis, then reduced and acetylated. The resulting alditol acetates were analyzed by gas chromatography coupled to mass spectrometry (GC-MS). Galactose, muramic acid and glucosamine were found almost exclusively in F2 fractions, only traces being detected in the F1 fractions (Fig. 4). Although most of the arabinose was also found in F2, significant amounts of this sugar were also observed in F1 fractions, consistent with the presence of lipoarabinomannan in these fractions (see below). DAP, another PG marker, was detected by GC-MS analysis after strong acid hydrolysis of the fractions, and followed by derivatisation of the products. DAP was abundant in the F2 fractions and, expectedly, only traces were detected in F1 fractions (Fig. 4). These data demonstrated that F2 fractions corresponded to the MMCW and that the fractions F1 were not contaminated by F2. For clarity, the two membranes F1 and F2 fractions were called thereafter PM and MMCW, respectively.

**Figure 5:**
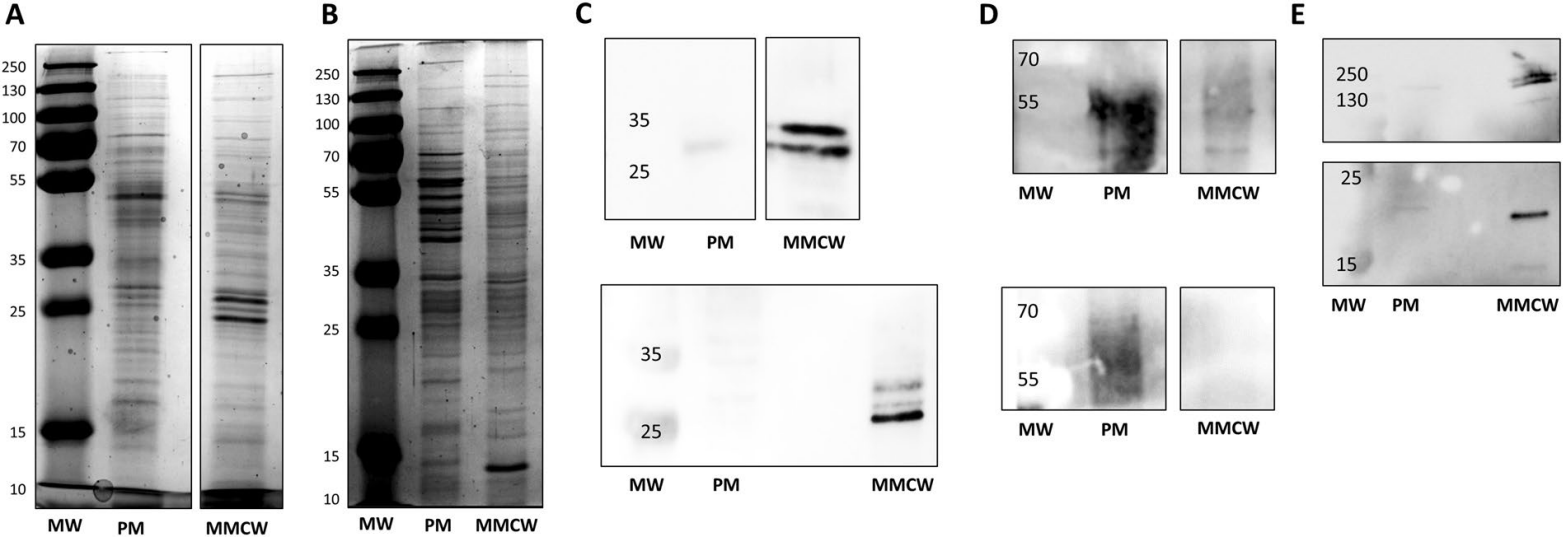
Analysis of mycobacterial membrane proteins. SDS-PAGE analysis of proteins extracted from the F1 (PM) and F2 (mycomembrane-containing cell walls, MMCW) of *Mau* (**A**) and *Msm* (**B**). Western blotting of PM and MMCW fractions from *Mau* (upper panel) and *Msm* (lower panel) using anti-Antigen85 (**C**) and anti-ATP synthase beta, AtpD (**D**) antibodies. Western blotting of PM and MMCW fractions from *Msm* using anti-MspA antibodies (**E**). MW: molecular weight markers in kDa.

The protein profile of each gradient fraction was determined by SDS-PAGE. The PM and MMCW fractions clearly showed distinctive patterns, suggesting a differential distribution of proteins within both types of membranes (Fig. 5A,B), and a few of them were identified by Western blot analysis. As no straightforward enzymatic test exists to specifically characterize the MM-bound proteins, we checked the fractions by immunodetection methods for the presence of very well characterized antigenic proteins known to be localized in the CW, namely, the mycoloyl transferases (the so-called antigen 85, Ag85)^23^. These enzymes catalyze the transfer of a mycoloyl residue from the trehalose monomycolate (TMM) to another TMM molecule to form trehalose dimycolate (TDM)^24^, or to arabinosyl residues of AG to form mycoloylated arabinosyl extremities^25–27^. Using anti-Ag85 polyclonal antibodies^28^, we specifically identified the Ag85 protein family in the MMCW, without residual signal in the PM, of both *Mau* and *Msm* (Fig. 5C). In *Msm*, the MspA protein has been characterized as the major porin for hydrophilic solutes uptake^16,29,30^, and is thus one of the very few specific proteins whose location in the MM is ensured. Anti-MspA antibodies revealed two bands in the *Msm* MMCW, one of low mass, around 20 kDa, and the second one at about 100 kDa, likely corresponding to the monomeric and octameric forms of MspA, respectively (Fig. 5E); expectedly, MspA was not detected in the PM of *Msm*. Finally, we checked for the presence of canonical PM proteins in both membrane fractions. Antibodies directed against the β-subunit of the membrane ATP synthase, AtpD (at around 52 kDa), indicated the presence of the enzyme in the PM fractions of both bacteria, while it was not detected in the MMCW fractions (Fig. 5D).

**Figure 6:**
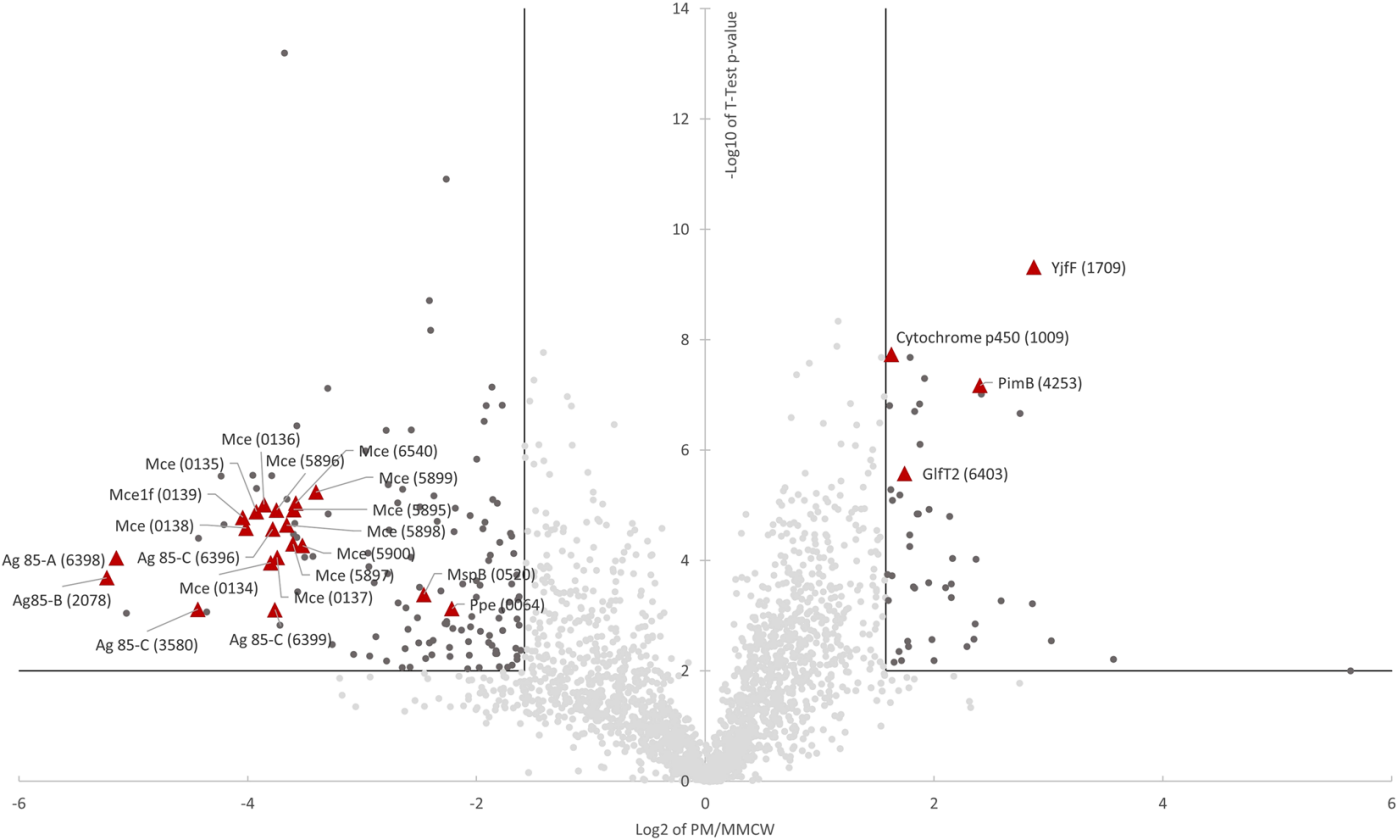
Quantitative proteomics analysis of *M. smegmatis* proteins. Volcano plot presentation of the statistical significance of *Msm* protein abundances as a function of protein abundance ratios between PM and MMCW fractions. Horizontal lines depict a p < 0.01 cutoff and vertical lines depict 1/3- and 3-fold ratios cutoffs. Each dot corresponds to a single identified protein. Dark grey dots indicate proteins enriched in each fraction. Proteins considered as markers of each membrane fraction and significantly enriched are represented in the volcano plot with red triangles. The corresponding protein name and gene number in parentheses are indicated.

Bottom-up quantitative proteomics study was performed on the PM and MMCW of the sequenced *M. smegmatis* mc² 155, in order to determine their protein contents and to evaluate the distribution of proteins within the two fractions. Accordingly, trypsin digestion was performed on each fraction concentrated in one band in a SDS-PAGE, and the resulting peptides mixtures were analyzed by nanoliquid chromatography coupled to tandem mass spectrometry (nanoLC-MS/MS) using an Orbitrap mass spectrometer. Then, database search and label free quantification allowed us to observe a different distribution of more than 2,100 proteins based on their intensity fold change between the PM and MMCW, as shown in the volcano plot (Fig. 6). The distribution profile of proteins between the two fractions unambiguously showed that they correspond to two different well-separated entities. The proteomics study not only confirmed the presence of proteins known to be located in the PM or MMCW but also allowed the identification and localization of other proteins. As expected, Ag85 complex proteins and porins, *e.g*. MspB, were present in MMCW (Fig. 6). Of note, the putative transporters MCE protein family were mostly found in MMCW fraction. On the other hand, mannosyltransferase PimB^31^, galactofuranosyltransferase GlfT2^32^, cytochrome p450 and ABC transporter YjfF, were found to be most abundant in the PM fraction (Fig. 6). The lists of other identified proteins among outliers (with a <1/3 or >3 fold ratio and a p < 0.01) and their distribution between the PM and MMCW are given in Tables 1 and 2 (Supplementary information). As previously noted^21^, proteins known to be primarily located in the cytosol and trapped in the liposomes may still contaminate the purified membrane fractions.

### Lipid content of the membranes

**Figure 7:**
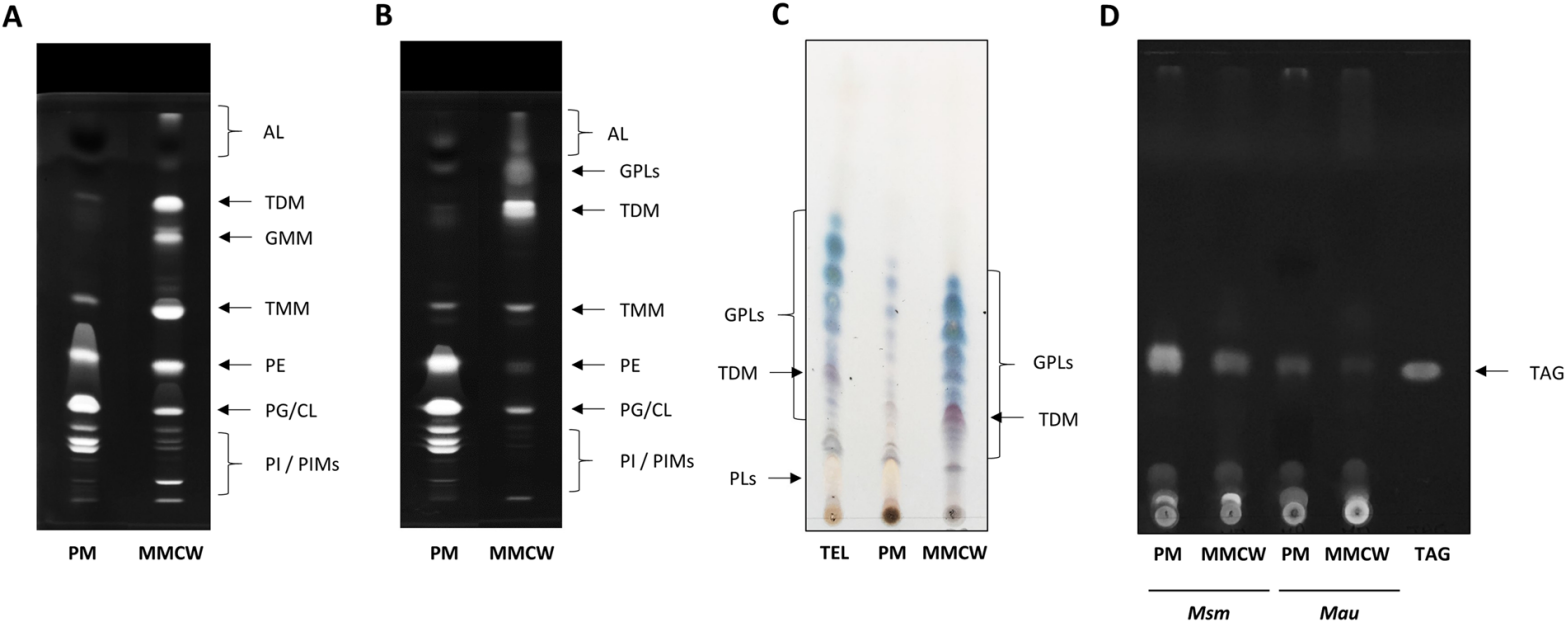
Analysis of mycobacterial membrane lipids. High performance thin-layer chromatography of lipids extracted from the PM and MMCW of *Mau* (**A**) and *Msm* (**B**). The plates were developed in CHCl_3_/CH_3_OH/H_2_O (65:25:4, v/v/v) and lipid spots were revealed by immerging the plate in primuline. In **C**: a TLC of lipids extracted from PM, MMCW and bacteria (TEL, total extractable lipids) of *Msm*. The plate was developed in CHCl_3_/CH_3_OH (9:1, v/v), and revealed with anthrone, followed by heating. The GPL are colored in blue on the plate. (**D**) Analysis of TAG: TLC were developed in Petroleum Ether /Diethyl Ether (9:1, v/v) and revealed by immerging the plate in a primuline bath. 100 µg of lipid mixtures were deposited on HPTLC plates. AL: apolar lipids; CL: cardiolopin; PG: phosphatidyl glycerol; PE: phosphatidyl ethanolamine; PI: phosphatidyl inositol; PIM: phosphatidyl-myo-inositol mannosides; TMM: trehalose monomycolates; TDM: trehalose dimycolates; GMM: glucose monomycolates; GPL: glycopeptidolipids; PL: phospholipids; TAG: triacylglycerols.

Lipids from each collected fraction were extracted with organic solvents and analyzed by using an automatized High-Performance-Thin-Layer Chromatography (HPTLC) quantitative system. Lipids were loaded on the silica gel plate, then separated in one dimension in an adapted solvent and the plates were sprayed with specific reagents that react with amino-compounds, glycoconjugates and phospholipids. Using purified standards, Rf values and reactivity to specific reagents, we identified each TLC spot corresponding to major lipids composing the MMCW and PM fractions. In both *Mau* and *Msm*, the PM showed the presence of glycerophospholipids, namely cardiolopin / phosphatidyl glycerol (CL/PG), phosphatidyl ethanolamine (PE), phosphatidyl inositol (PI) and phosphatidyl-myo-inositol mannosides (PIM). In addition, small amounts of TMM, a compound known to be a precursor of TDM, were also observed in these fractions (Fig. 7A,B). However, TDM was completely absent from PM, consistent with and reinforcing the fact that there is no significant contamination of PM by MMCW constituents.

The MMCW fractions contained important amounts of trehalose mycolates, *i.e*. TDM and TMM, plus glucose monomycolates (GMM) in the case of *Mau* F2 (Fig. 7A), due to the presence of glucose as carbon source. In addition to TMM and TDM, significant amounts of phospholipids (CL/PG, PE and PI, plus some PIM) were also detected in the F2 fractions, notably that of *Mau*, in contrast to *Cgl* ^21^. To ascertain the presence of phospholipids in the MMCW fractions, as opposed to a contamination origin, we reasoned that the amount of lipids attached to the AG-PG and forming the inner leaflet of the MM (exclusively MA), would be equal to that present in the outer leaflet if the phospholipids observed derived from contamination. Accordingly, we quantified the MA attached to AG-PG and those esterifying trehalose (TMM and TDM), which are extractable with organic solvents. In *Mau*, in which large amounts of phospholipids were present in the MMCW, the amount of MA released by saponification of the fraction (2.95% of dry cell weight +/− 0.16%) was 4-times higher than that found in the extractable lipids (derived from TMM and TDM). This data indicated that other lipids, in addition to TDM and TMM, participate to the outer leaflet of MM of *Mau*. Consistently, the ratio of MA linked to AG-PG *versus* MA linked to trehalose was 1.6 for *Msm*, in which less phospholipids were present in the MMCW fraction. Besides, the MA composition of both membrane fractions were identical. The three expected types of mycolates (derived from the saponification of PM and MMCW followed by methylation) were identified by TLC for both species: α-, keto- and dicarboxy-MA, for *Mau*, and α-, α’- and epoxy-MA for *Msm*, in both PM and MMCW.

The type-species specific glycopeptidolipids (GPL) were expectedly found most exclusively in the MMCW of *Msm* (Fig. 7C). These lipids have been previously shown to be located on the bacterial surface and in deeper compartment of the cell envelope^15,33^. The less polar lipids, which represent minor components of the two membrane fractions from the two mycobacterial species (Fig. 7A,B), were composed, as least partly, of triacylglycerols (TAG, Fig. 7D). However, examination of the lipid content of a fraction exhibiting the lowest density, at the top of all sucrose gradients from the mycobacterial lysates examined, showed that it consists of TAG, probably derived from the cytosolic lipid droplets. Consequently, although TAG has been identified in the lipids extracted from the outermost compartments of mycobacterial cell envelope^15^, the TAG found in the PM and MMCW of both *Mau* and *Msm* may originate from the cytosol.

### Lipoglycan localization

**Figure 8:**
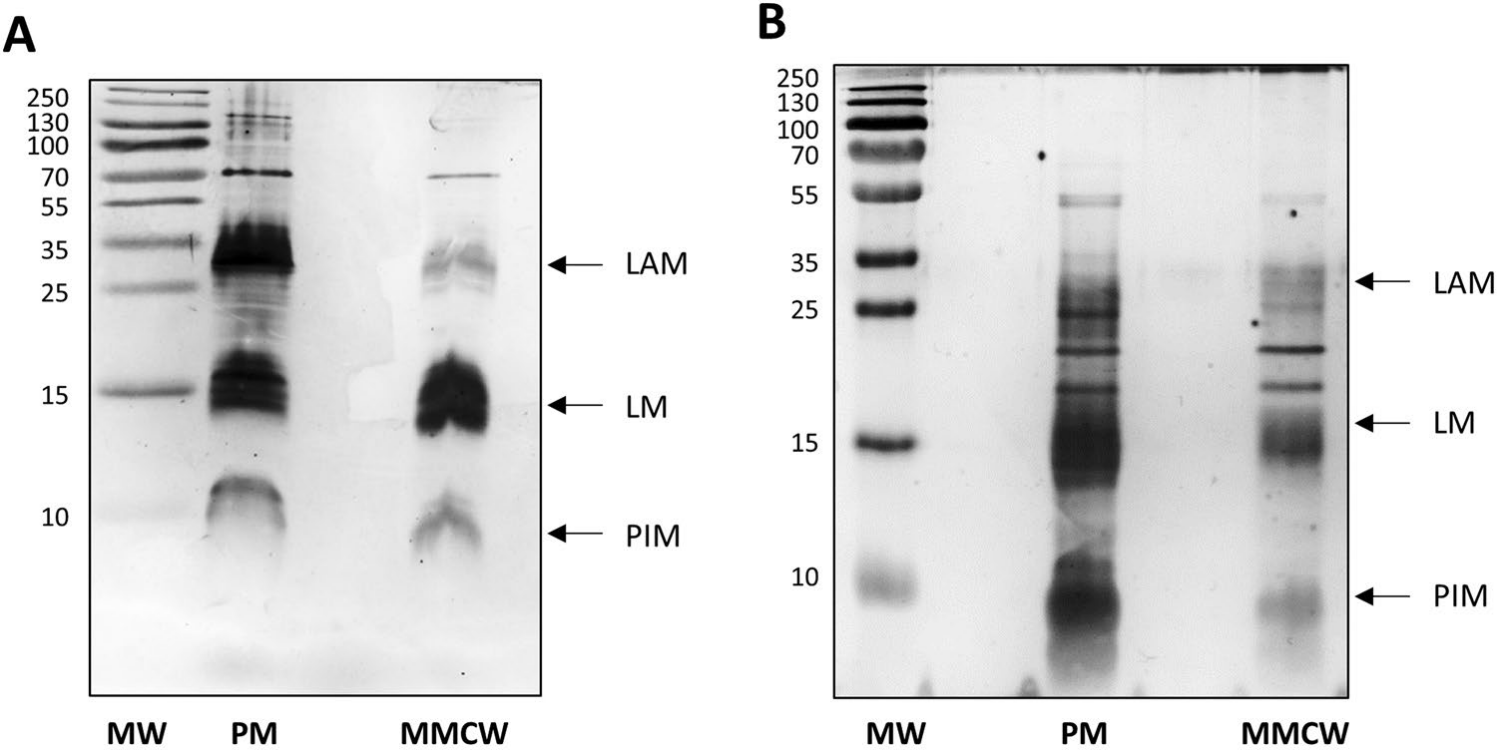
Analysis of mycobacterial membrane lipoglycans. SDS-PAGE analysis of lipoglycans from the PM and MMCW of *Mau* (**A**) and *Msm* (**B**). F1 and F2 fractions were digested with proteases prior to their analysis. LAM: lipoarabinomannan; LM: lipomannan; PIM: phosphatidyl-myo-inositol mannosides. MW: molecular weight markers in kDa.

PIM, lipomannans (LM) and lipoarabinomannans (LAM) are glycoconjugates of the cell envelope but their exact localization still is a matter of debate^3^. A dramatic reduction of the LAM content on stationary phase in *Msm* has been reported, suggesting that its synthesis is selectively modulated by the growth phase^34^. We checked for the presence of lipoglycans in the two membrane fractions of both *Mau* and *Msm* using SDS-PAGE after trypsin digestion of each fraction. PIM, LM and LAM were observed in both the MMCW and PM fractions of the two mycobacteria examined (Fig. 8). In both species, the amount of LAM was considerably lower in the MMCW than in the PM. Interestingly, in the stationary growth phase, the LAM content of *Mau* decreased dramatically in both PM (3.4-fold) and MMCW (2.4-fold) (Fig. S1). This may be due either to a response of cells to growth change, to adapt their membrane composition or, alternatively, to the action of putative endomannanase to generate arabinomannans, known to be present in the outermost compartment of mycobacterial cells^13^.

## Discussion

The central role of the mycobacterial cell envelope in essential physiological processes makes the determination of its composition and arrangement of its constituents an important field of research. Presently, these remain unproven and deduced either from its global lipid content or from the analysis of detergent-extracted materials. Accordingly, we addressed these questions by isolating the native MM-containing CW (MMCW) and PM from two mycobacterial model species, *Mau* and *Msm*. Both species are non-pathogenic rapid-growing mycobacteria, and present an outermost layer^35^. Our choice was based on the fact that *Mau* is the only mycobacterial species that exposes TDM at its cell surface^15^, suggesting that no polymeric (capsular) substances cover its cell wall. Moreover, this strain was largely used in laboratories for studies concerning isoniazid and ethionamide, two antituberculous agents^36–38^. *Msm* is commonly used and validated as a surrogate study model for slow growing pathogenic mycobacteria and its genome sequence is available, facilitating comparison with sequenced mycobacterial pathogens.

We have previously compared different methods for lysis and fractionating mycobacteria cells^20^ and subsequently have succeeded in isolating and characterizing the pure MMCW from a bacterium of the phylogenetically related *Corynebacterium* genus, namely *Cgl*, using separation of membrane fractions by isopycnic sucrose gradient centrifugation^21^. We thus combined various methods for isolating, for the first time, the MMCW of *Mau*. The purity of subcellular fractions was monitored by using specific PM and MMCW markers that showed that they were virtually free from significant contamination by other cell fractions. The isolation of equivalent fractions from *Msm* was, however, more difficult to achieve. The sucrose step gradient optimized for *Mau* was poorly effective to separate the two fractions of *Msm* and the use of several degradative enzymes to hydrolyze AG and PG and detergents to extract and/or remove non-specific interacting surface-exposed compounds were unsuccessful. Nevertheless, the use of an amended sucrose gradient yielded two homogeneous fractions from *Msm*. PM fractions from both mycobacterial species devoid of specific CW markers, namely DAP, muramic acid and glucosamine, were obtained by isopycnic sucrose gradient centrifugation of lysates. Similarly, MMCW fractions were isolated from both *Mau* and *Msm*, in which PM enzymatic markers, *i.e*. NADH oxidase and ATP synthase (subunit beta) were not detected by enzymatic assay and western blotting respectively. Immuno-detection by Western blotting identified the well characterized antigenic proteins, the so-called antigen 85^23^in the MMCW fractions but not in the PM. Similarly, the major porin for hydrophilic solutes uptake of *Msm*, MspA^16,29,30^, was specifically localized in the MMCW fraction, but not detected in *Msm* PM. The fractions were further characterized by bottom-up quantitative proteomics study to assess the protein content and its distribution between the two membrane fractions. These analyses confirmed the occurrence of proteins known to be located in the PM or MMCW. Moreover, other proteins were identified such as the putative transporters MCE protein family mostly located in the MMCW fraction and PimB, GlfT2, cytochrome P450 and the inner membrane ABC transporter YjfF, mostly present in PM fraction, as expected.

The inner leaflet of the MM is very likely formed by a parallel arrangement of MA (Fig. 1) whereas its outer leaflet is presumably composed of various lipids whose composition is a matter of debate. This is due to the diversity of methods used for determining the MM composition. We thus took the opportunity of having native MMCW and PM to address the question of their lipid compositions. Lipids from the purified PM and MMCW fractions from the two mycobacterial species were analyzed by HPTLC. Expectedly, phospholipids represented the major lipids of the PM. They were also present in significant amounts in the MMCW fractions, notably that of *Mau*, composed primarily of trehalose mycolates, namely TDM and TMM, and GMM. The real occurrence of phospholipids in the MMCW fractions was demonstrated by quantifying the MA. The amounts of MA covalently-linked to the AG was 2- to 4-times higher than those found in the extractable lipids of the two mycobacteria, indicating that other lipids, in addition to trehalose mycolates, participate to the outer leaflet of MM. Interestingly, no phospholipid was detected in the MMCW fraction of *Cgl* ^21^, reinforcing the concept that this class of lipids is really constitutive of mycobacterial MM. With the native PM and MMCW in hands, we looked for the presence of lipoglycans in the two membrane fractions. Their localization, notably that of LAM, is still a matter of debate. Again, the methods used are crucial for a proper localization of compounds. Although their abundance may vary, according to the growth phases, PIM, LM and LAM were all observed in both the MMCW and PM fractions of the two mycobacteria examined, *Mau* and *Msm*, the PM being, in both species, the main location of LAM.

Our data on the composition of the native outer membrane, the MM, of *Msm* differ from that of Bansal-Mutalik and Nikaido using detergent-extraction, namely dioctylsulfosuccinate sodium^10^, in several points. Firstly, we showed that the non-covalently bound lipids, presumably constituting the outer leaflet of the MM-containing CW of the two species examined, are composed mainly of TDM and TMM, lipoglycans and phospholipids, and the PM is conventionally composed of phospholipids and proteins. TMM was located by Bansal-Mutalik and Nikaido in the PM using the detergent-extraction method^10^. Although TMM was found in the present study in the PM, this is interpreted as molecules transported across the PM to its final destination, *i.e*. MMCW (Fig. 1). Importantly, the occurrence of TMM has never been reported in isolated mycobacterial PM^3^. Rather, TMM have been isolated from the cell surface of several mycobacterial species by mechanical treatment^15^. Secondly, we have previously localized glycopeptidolipids (GPL) at the mycobacterial cell surface, representing half of the bacterial GPL. The remaining GPL were present in deeper compartments of the cell envelope^33^. Consistently, GPL were found in the MMCW fraction of *Msm* (Fig. 1). Bansal-Mutalik and Nikaido have proposed that GPL locate in the MM, which is crucial for the mycobacterial life, as demonstrated by the non-viability of strains in which the production of MA is abolished^39,40^. As strains devoid of GPL were found viable, displaying no significant physiological changes, notably in terms of growth rate, with an apparent intact MM^33^, GPL, and by extension other lipids of the outer leaflet of the MM, are likely dispensable, unlike MA for the existence of the MM. Finally, Bansal-Mutalik and Nikaido proposed TAG as constituents of the MM. Although consistent with our finding, the isolation of a TAG-containing fraction on the top of all sucrose gradients of mycobacterial lysates, suggests that these molecules probably derived from the cytosolic lipid droplets. Furthermore, inserting these apolar lipids in a biological membrane is hard to conceive. Based on the identification of TAG in the lipid extracted from the outermost compartments of mycobacterial cell envelope^15^, we propose that TAG are part of surface lipids that form, with carbohydrates and proteins in a yet unknown manner, the OL above the MM.

Several questions remain to be solved regarding the MM. Among these, the isolation of the MM, instead of the more complex and less practicable MMCW. This would need the use of degradative enzymes to extract as much as possible the AG-PG complex and the loosely bound polysaccharides and proteins, combined with mechanical treatments and/or extraction with very low concentrations of Tween-80^13,15^ these experiments are in progress. Another important issue is the occurrence and nature of proteins other than porins, and the arrangement of MM constituents. Nevertheless, with the progress in the knowledge of the MMCW composition, it will now be possible to study the spatial organization and structure of the MM, which will very likely mark a major evolution for the cell envelope structure and the biology of mycobacteria. The discovery of how mycobacteria select and organize their membrane constituents to resist killing should help deciphering the mechanisms involved in the mycobacterial pathogenicity and provide new potential targets for tuberculosis chemotherapy.

## Methods

### Bacterial strains and growth conditions

*Mycobacterium aurum* (*Mau*) A + (CIP104482) was grown in 7H9 Middlebrook broth (BD Difco) supplemented with casitone (0.5%; BD Difco) and glucose (0.2%) at 37 °C under shaking (180 rpm). *M. smegmatis* (*Msm*) mc²155 was grown in 7H9 Middlebrook broth (BD Difco) medium supplemented with glycerol (0.2%; Sigma) at 37 °C under shaking (180 rpm).

### Bacteria lysis and fractionation on sucrose density gradient

Cells were harvested by centrifugation at 3,000 *g* for 15 min at 4 °C and the cell pellet was washed with 20 mM Tris HCl, pH 7.4. The pellet was frozen and cells were suspended in the lysis buffer (20 mM Tris HCl, pH 7.4 containing benzonase (5 µL > 250 units/mL, Sigma-Aldrich), dithiothreitol (1 mM), AEBSF (0.2 mM; Euromedex) and EDTA (1 mM)). After 20 min, bacteria were broken by two passages through a French press cell (1,500 bars). Unbroken bacteria were removed by centrifugation (3,000 *g* for 10 min, twice). The bacterial lysate was then submitted to centrifugation at 10,000 *g* for 40 min to yield crude cell wall fraction (pellet P10). P10 was suspended in 20 mM Tris HCl, pH 7.4 containing EDTA (1 mM) and layered on a sucrose step gradient (from 10% w/w to 60% w/w) in SW41 tubes (Beckman Coulter). The discontinuous gradient consisted of 10% [1 Vol] – 36% [3 Vol] – 40% [3 Vol] – 42% [2 Vol] – 50% [1 Vol] – 60% [1 Vol], in the case of *Mau*. It was amended (10% [1 Vol] – 20% [1 Vol] – 30% [3 Vol] – 36% [3 Vol] – 40% [1 Vol] – 50% [1 Vol] – 60% [1 Vol]) to achieve a well separation of the two membrane fractions of *Msm*. The gradients were centrifuged for at least 2 h at 100,000 *g*. Fractions of 1 mL were collected from the bottom to the top, washed and the pellets were resuspended in the same buffer, layered on sucrose gradient and centrifuged again in the same conditions. Fractions of 1 mL were collected from the bottom to the top, washed and recentrifuged (15,000 *g*, 45 min) to recover the cell walls, which were submitted to biochemical analyses. The supernatant (S10) was then centrifuged at 27,000 *g* for 30 min and the pellet (P27) was discarded. The S27 supernatant was then centrifuged at 100,000 *g* for 40 min to collect the crude plasma membrane (pellet P100) (Fig. 2). P100 was suspended in 20 mM Tris HCl, pH 7.4 containing EDTA (1 mM) before being layered on a sucrose step gradient (from 10% w/w to 60% w/w) in SW41 tubes (Beckman Coulter). The gradients were centrifuged for at least 2 h at 100,000 *g*. Fractions of 1 mL were collected from the bottom to the top, washed and membrane pellets were recovered after centrifugation (100,000 *g*, 1 h) and submitted to biochemical analyses. All these steps were done at 4 °C.

### Protein analysis

Proteins were quantified using the DC Protein assay Kit (Biorad) and separated on SDS-PAGE (stacking 5%, resolving 12% acrylamide). For Western blotting, proteins were separated on SDS-PAGE minigel (stacking 5%, resolving 12% acrylamide) and transferred on nitrocellulose membrane (0.45 µm) using trans-blot turbo blotting system (Biorad). Membranes were blocked with TBS containing milk (5%), then blotted with primary antibodies αMsp A (rabbit), αATP synthase beta (chicken) from Abcam, αHyt 27 (mouse) diluted in TBS containing BSA (5%). After washes with TBS containing Tween 20 (0.02%), secondary antibodies (αrabbit-HRP (goat) from Santa Cruz Biotech, αchicken-HRP (goat) from Abcam and α-mouse-HRP (goat) from Biorad) were incubated for 1 h. After washes, chemiluminescence was detected using the Chemidoc Touch Images (Biorad).

### Quantitative proteomics analysis

Proteomics analyses were performed on biological duplicates of *Msm* PM and MMCW fractions. Preparation of the protein samples for MS analysis is described in the Supplemental Information. Briefly, after a concentration step on SDS-PAGE, proteins from each fraction were in-gel digested using trypsin. Then, the resulting peptides were extracted from the gel and analyzed in triplicate injections by nanoLC-MS/MS using an Orbitrap mass spectrometer. MS data were searched against the Uniprot *M. smegmatis* mc² 155 database for protein identification and label-free quantification was performed to compare protein abundances. See the Supplemental information section for further details.

### Enzymatic activity detection

NADH oxidase activity was measured *via* the DCPIP reduction at 608 nm. Enzymatic reactions were performed at room temperature for at least 1 min in 96-wells plate. 50 µL of membrane samples containing the same amount of proteins were incubated in 200 µL of 100 mM Tris-HCl (pH 7.4), MgCl_2_ (5 mM), CaCl_2_ (10 mM), NaN_3_ (45 mM), NADH (0.25 mM) and DCPIP (12.5 µM). The kinetic measurements were made with Clariostar spectrophotometer (BMG Labtech) and data were obtained with the Clariostar Mars software (BMG Labtech). The values of the slopes were directly correlated to the reaction velocities.

### Lipid analysis

Portions (400 µL) of the harvested fractions were submitted to a Bligh and Dyer extraction at room temperature^41^. Briefly, 0.8 V of each gradient fraction was incubated in one phase solvent system CHCl_3_/CH_3_OH (1:2, v/v) for 16 h at room temperature. Then, CHCl_3_ and H_2_O (1:1, v/v) were added to obtain two phases and organic lower phases were dried under nitrogen. Then, 100 µg of each lipid fraction (10 mg.mL^−1^ in CHCl_3_) were analyzed by HPTLC. Merck HPTLC silica gel 60 was developed in CHCl_3_/CH_3_OH/H_2_O (65:25:4, v/v/v) (CAMAG). Lipids were revealed with primuline (0.01% in acetone/water (8:2)) and the plate scanned at 370 nm. Data were treated with Wincats software and lipids were relatively quantified.

### Mycolic acid analysis

Gradient fractions were saponified with 2 mL of KOH (40%)/2-methoxyethanol (1:7; v/v) for 3 h at 110 °C in a screw capped tube^42^. The solutions were acidified by adding sulfuric acid (20%) to reach a pH 1~2. Mycolic acids were extracted 3 times with diethyl ether and the organic phases were combined. After drying, the resulting fatty acids were methylated using diazomethane and analyzed by HPTLC with CH_2_Cl_2_ as developing solvent. Visualization was realized by immersion of the plate in a 10% CuSO_4_ (w/v) solution in H_3_PO_4_/CH_3_OH/H_2_O (8:5:87, v/v/v) followed by heating at 150 °C, 20 min.

For quantifying MA, lipids were extracted from wet cells for 16 h with CHCl_3_/CH_3_OH (1:2, v/v) at room temperature (60 mL of solvent/g of wet cells), then with CHCl_3_/CH_3_OH (1:1, v/v) and finally with CHCl_3_/CH_3_OH (2:1, v/v). The organic phases were pooled, concentrated and washed with water, resulting in the total extractable lipids. MA, from total extractable lipids or from delipidated residues (containing AG-bound MA) were saponified and treated as previously described^42^. MA were separated from fatty acids by column chromatography on Florisil using a petroleum ether/diethylether step gradient (0, 5, 10, 20% and 100%, v/v). Each fraction was analyzed by TLC developed with CH_2_Cl_2_. MA were detected by spraying plates with phosphomolybdic acid (10% w/v diluted in ethanol), followed by heating. Fractions containing MA were pooled and weighed.

### Lipopolysaccharide analysis

Lipoglycans (LAM, LM and PIM) were analyzed by SDS-PAGE (stacking 5% and resolving 15% acrylamide). Samples, containing the same amount of proteins, were digested by trypsin (Promega) at 37 °C overnight before the addition of the loading buffer. Gels were stained with silver staining^43,44^. Gels were scanned with Chemidoc Touch Images (Biorad) and analysed with Image Lab.

### Analysis of diaminopimelic acid content

Washed and dried gradient fractions were hydrolyzed with 6 M HCl (500 µL) at 110 °C overnight. After cooling, the hydrolysates were centrifuged at 1,600 *g* for 20 min. Supernatants were dried under nitrogen and esterified with 2.5 M HCl in 2-propanol (500 µL) for 1 h at 110 °C. After drying under nitrogen, a treatment with trifluoroacetic anhydride (50 µL in 300 µL CH_2_Cl_2_) was done 1 h at 110 °C^45^. After drying under nitrogen, petroleum ether (1 mL) was added and 10 µL of samples were analyzed by GC-MS.

### Sugar analysis

Washed and dried gradient fractions were hydrolyzed with 2 N CF_3_COOH at 110 °C for 2 h, dried under nitrogen and reduced with NaBH_4_ (10 mg/mL in 1 N NH_4_OH/CH_3_CH_2_OH 1:1, v/v) at room temperature for 2 h. The reaction was stopped with few drops of acetic acid, dried under nitrogen, and acetylated with pyridine anhydrous/acetic anhydride (1:1, v/v) at room temperature overnight. After drying under nitrogen, petroleum ether (1 mL) was added and 1 µL of samples were analyzed by GC-MS. GC-MS analyses were performed using a Thermo TraceGCultra chromatograph equipped with an Inferno ZB5HT column (30 m × 0.25 mm) and connected to an ISQ single quadrupole mass <220 °C with split ratio of 20:1. Helium circulates at a constant flow rate of 1.2 mL.min^−1^ as carrier gas. The temperature separation program was: initial temperature at 100 °C and then increased until 300 °C at a rate of 20 °C min^−1^, followed by 3 min at 300 °C.

### Transmission electron microscopy

Specimens were prepared for electron microscopy using the conventional negative staining procedure. 20 μL of solution was absorbed on Formvar-carbon-coated grids for 2 min, blotted, and negatively stained with uranyl acetate (1%) for 1 min. Grids were examined with a TEM (Jeol JEM-1400, JEOL Inc, Peabody, MA, USA) at 80 kV. Images were acquired using a digital camera (Gatan US1000, Gatan Inc, Pleasanton, CA, USA) at different magnifications: × 3,000 for MMCW fractions, × 12,000 for PM *Msm* and × 25,000 for PM *Mau*.

## Electronic supplementary material

Supplementary Information
