# Supplementary material for: Dissecting the mycobacterial cell envelope and defining the composition of the native mycomembrane
Source: Sci Rep. 2017 Oct 9;7:12807. doi: 10.1038/s41598-017-12718-4 (PMC5634507; doi:10.1038/s41598-017-12718-4)

Supplementary information

Title: Dissecting the mycobacterial cell envelope and defining the composition of the native mycomembrane

Authors:

Laura Chiaradia[1,2], Cyril Lefebvre[1,2], Julien Parra[1,2], Julien Marcoux[1,2], Odile Burlet-Schiltz[1,2], Gilles Etienne[1,2], Maryelle Tropis[1,2]* and Mamadou Daffé[1,2]*

Protein digestion and nanoLC-MS/MS analysis

25 µg of each protein sample was reduced by incubation with DTT (25 mM) at 95°C, and then alkylated with iodoacetamide (100 mM) for 30 min at room temperature in the dark. Samples were then loaded and concentrated on a SDS-PAGE. For this purpose, the electrophoresis was stopped as soon as the proteins left the stacking gel to enter the resolving gel as one single band. The proteins, revealed with Instant Blue (Expedeon) for 20 minutes, were found in one blue band of around 5 mm width. The band of each fraction was cut and washed before the in-gel digestion of the proteins overnight at 37°C with a solution of modified trypsin. The resulting peptides were extracted from the gel using two successive incubations in 10 % formic acid (FA)/acetonitrile (ACN) (1:1, v/v) for 15 min at 37°C. The two collected fractions were pooled, dried and resuspended with 25 µL of 2 % ACN, 0.05 % trifluoroacetic acid (TFA). NanoLC-MS/MS analysis was performed in triplicate injections using an Ultimate 3000 nanoRS system (Dionex) coupled to an LTQ-Orbitrap Velos mass spectrometer (Thermo Fisher Scientific) operating in positive mode. 5 µL of each sample were loaded onto a C18-precolumn (300 µm inner diameter x 5 mm) at 20 µL/min in 2 % ACN, 0.05 % TFA. After 5 min of desalting, the precolumn was switched online with the analytical C18 nanocolumn (75 µm inner diameter x 15 cm, packed in-house) equilibrated in 95 % solvent A (5 % ACN, 0.2 % FA) and 5 % solvent B (80 % ACN, 0.2 % FA). Peptides were eluted by using a 5-25 % gradient of solvent B for 75 min, then a 25-50 % of solvent B for 40 min at a flow rate of 300 nL/min. The LTQ-Orbitrap Velos was operated in data-dependent acquisition mode with the XCalibur software. Survey scans MS were acquired in the Orbitrap, on the 300-2,000 *m/z* (mass to charge ratio) range, with the resolution set to a value of 60,000 at *m/z* 400. Up to twenty of the most intense multiply charged ions (2+ and 3+) per survey scan were selected for CID fragmentation, and the resulting fragments were

analysed in the linear ion trap (LTQ). Dynamic exclusion was used within 60 s to prevent repetitive selection of the same peptide.

Bioinformatic MS data analysis

Mascot (Mascot server v2.6.0; http://www.matrixscience.com) database search engine was used for peptide and protein identification using automatic decoy database search to calculate a false discovery rate (FDR). MS/MS spectra were compared to the UniProt *M. smegmatis* strain ATCC 700084 / mc^2 155 database (release June 2017, 12,683 sequences). Mass tolerance for MS and MS/MS was set at 8 ppm and 0.6 Da, respectively. The enzyme selectivity was set to full trypsin with two missed cleavages allowed. Protein modifications were fixed carbamidomethylation of cysteines, variable oxidation of methionine, variable acetylation of protein N-terminus. Proline software was used for the validation and the label-free quantification of identified proteins in each sample (http://proline.profiproteomics.fr/). Mascot identification results were imported into Proline. Search results were validated with a peptide rank=1 and at 1 % FDR both at PSM level (on Adjusted e-Value criterion) and protein sets level (on Modified Mudpit score criterion). The identification summaries (*i.e.*, the validated Mascot search results) of all the MS analyses corresponding to one biological replicate were merged and a protein inference list was created. Label-free quantification was performed for all protein identified: peptides are quantified by extraction of MS signals in the corresponding raw files, and post-processing steps can be applied to filter, normalize, and aggregate peptide quantitative data into protein abundance values. Proteins with more than 3 missing values per fraction were excluded.

Proteins of *Msm* enriched in PM and MMCW fractions.

Table 1: List of proteins of *Msm* significantly enriched in the MMCW fraction.

Proteins of *Msm* identified by quantitative proteomics analyses as specifically enriched in the MMCW fraction as compared to the PM fraction (ratio PM/MMCW < 1/3 and p-value < 0.01) are listed. The MS intensity ratios are expressed in Log_2 and the p-values are expressed in $-Log_{10}$.

Accession	Gene Name	Ratio	P-Value	Protein Name
**sp|A0QU51|A85B_MYCS2**	fbpB	-5,231	3,691	Diacylglycerol acyltransferase/mycolyltransferase Ag85B OS=Mycobacterium smegmatis (strain ATCC 700084 / mc(2)155) GN=fbpB PE=2 SV=1
**tr|A0R623|A0R623_MYCS2**	MSMEG_6398	-5,148	4,047	Antigen 85-A OS=Mycobacterium smegmatis (strain ATCC 700084 / mc(2)155) GN=MSMEG_6398 PE=4 SV=1
**tr|A0QUY5|A0QUY5_MYCS2**	MSMEG_2381	-5,063	3,047	Uncharacterized protein OS=Mycobacterium smegmatis (strain ATCC 700084 / mc(2)155) GN=MSMEG_2381 PE=4 SV=1
**tr|A0QY95|A0QY95_MYCS2**	MSMEG_3580	-4,434	3,12	Antigen 85-C OS=Mycobacterium smegmatis (strain ATCC 700084 / mc(2)155) GN=MSMEG_3580 PE=4 SV=1
**tr|A0QQ67|A0QQ67_MYCS2**	MSMEG_0645	-4,432	4,41	Putative beta-1,3-glucanase OS=Mycobacterium smegmatis (strain ATCC 700084 / mc(2)155) GN=MSMEG_0645 PE=4 SV=1
**tr|A0R2T3|A0R2T3_MYCS2**	MSMEG_5225	-4,36	3,07	Uncharacterized protein OS=Mycobacterium smegmatis (strain ATCC 700084 / mc(2)155) GN=MSMEG_5225 PE=4 SV=1
**tr|A0QW33|A0QW33_MYCS2**	MSMEG_2790	-4,233	5,537	Uncharacterized protein OS=Mycobacterium smegmatis (strain ATCC 700084 / mc(2)155) GN=MSMEG_2790 PE=4 SV=1
**tr|A0QVP4|A0QVP4_MYCS2**	MSMEG_2645	-4,21	4,652	Uncharacterized protein OS=Mycobacterium smegmatis (strain ATCC 700084 / mc(2)155) GN=MSMEG_2645 PE=4 SV=1
**tr|A0QNR7|A0QNR7_MYCS2**	MSMEG_0139	-4,043	4,782	MCE-family protein MCE1f OS=Mycobacterium smegmatis (strain ATCC 700084 / mc(2)155) GN=MSMEG_0139 PE=4 SV=1
**tr|A0QNR6|A0QNR6_MYCS2**	MSMEG_0138	-4,014	4,589	Virulence factor Mce family protein OS=Mycobacterium smegmatis (strain ATCC 700084 / mc(2)155) GN=MSMEG_0138 PE=4 SV=1
**tr|A0QY10|A0QY10_MYCS2**	MSMEG_3494	-3,956	5,55	Putative secreted protein OS=Mycobacterium smegmatis (strain ATCC 700084 / mc(2)155) GN=MSMEG_3494 PE=4 SV=1

**tr|A0R526|A0R526_MYCS2**	MSMEG_6040	-3,925	5,308	Uncharacterized protein OS=Mycobacterium smegmatis (strain ATCC 700084 / mc(2)155) GN=MSMEG_6040 PE=4 SV=1
**tr|A0QNR3|A0QNR3_MYCS2**	MSMEG_0135	-3,923	4,887	MCE-family protein MCE1b OS=Mycobacterium smegmatis (strain ATCC 700084 / mc(2)155) GN=mce2B PE=4 SV=1
**tr|A0QNR4|A0QNR4_MYCS2**	MSMEG_0136	-3,857	5,016	MCE-family protein MCE1c OS=Mycobacterium smegmatis (strain ATCC 700084 / mc(2)155) GN=mce1C PE=4 SV=1
**tr|A0QNR2|A0QNR2_MYCS2**	MSMEG_0134	-3,798	3,963	MCE-family protein MCE1a OS=Mycobacterium smegmatis (strain ATCC 700084 / mc(2)155) GN=mce PE=4 SV=1
**tr|A0QP20|A0QP20_MYCS2**	MSMEG_0243	-3,792	5,544	Uncharacterized protein OS=Mycobacterium smegmatis (strain ATCC 700084 / mc(2)155) GN=MSMEG_0243 PE=4 SV=1
**tr|A0R621|A0R621_MYCS2**	MSMEG_6396	-3,781	4,569	Antigen 85-C OS=Mycobacterium smegmatis (strain ATCC 700084 / mc(2)155) GN=mpt51 PE=4 SV=1
**tr|A0R624|A0R624_MYCS2**	MSMEG_6399	-3,761	3,111	Antigen 85-C OS=Mycobacterium smegmatis (strain ATCC 700084 / mc(2)155) GN=fbpA PE=4 SV=1
**tr|A0R4N4|A0R4N4_MYCS2**	MSMEG_5896	-3,747	4,917	Virulence factor Mce family protein OS=Mycobacterium smegmatis (strain ATCC 700084 / mc(2)155) GN=MSMEG_5896 PE=4 SV=1
**tr|A0QNR5|A0QNR5_MYCS2**	MSMEG_0137	-3,742	4,058	MCE-family protein MCE1d OS=Mycobacterium smegmatis (strain ATCC 700084 / mc(2)155) GN=mce2D PE=4 SV=1
**tr|A0R4B1|A0R4B1_MYCS2**	MSMEG_5770	-3,72	2,831	Uncharacterized protein OS=Mycobacterium smegmatis (strain ATCC 700084 / mc(2)155) GN=MSMEG_5770 PE=4 SV=1
**tr|A0R0E0|A0R0E0_MYCS2**	MSMEG_4353	-3,679	13,2	Uncharacterized protein OS=Mycobacterium smegmatis (strain ATCC 700084 / mc(2)155) GN=MSMEG_4353 PE=4 SV=1
**tr|A0R4N6|A0R4N6_MYCS2**	MSMEG_5898	-3,66	4,646	MCE-family protein MCE4c OS=Mycobacterium smegmatis (strain ATCC 700084 / mc(2)155) GN=mce4C PE=4 SV=1
**tr|A0QY09|A0QY09_MYCS2**	MSMEG_3493	-3,659	5,119	Putative secreted protein OS=Mycobacterium smegmatis (strain ATCC 700084 / mc(2)155) GN=MSMEG_3493 PE=4 SV=1
**tr|A0R4N5|A0R4N5_MYCS2**	MSMEG_5897	-3,603	4,301	MCE-family protein MCE4d OS=Mycobacterium smegmatis (strain ATCC 700084 / mc(2)155) GN=MSMEG_5897 PE=4 SV=1
**tr|A0QTL5|A0QTL5_MYCS2**	MSMEG_1887	-3,601	4,484	Uncharacterized protein OS=Mycobacterium smegmatis (strain ATCC 700084 / mc(2)155) GN=MSMEG_1887 PE=4 SV=1
**tr|A0R4N3|A0R4N3_MYCS2**	MSMEG_5895	-3,598	4,927	MCE-family protein MCE4f OS=Mycobacterium smegmatis (strain ATCC 700084 / mc(2)155) GN=mce4F PE=4 SV=1

**tr|A0R722|A0R722_MYCS2**	MSMEG_6752	-3,59	4,68	Glucanase OS=Mycobacterium smegmatis (strain ATCC 700084 / mc(2)155) GN=MSMEG_6752 PE=3 SV=1
**tr|A0R6G4|A0R6G4_MYCS2**	MSMEG_6540	-3,58	5,04	MCE-family protein MCE1a OS=Mycobacterium smegmatis (strain ATCC 700084 / mc(2)155) GN=mce PE=4 SV=1
**tr|A0R4L6|A0R4L6_MYCS2**	MSMEG_5878	-3,573	4,424	Cutinase OS=Mycobacterium smegmatis (strain ATCC 700084 / mc(2)155) GN=MSMEG_5878 PE=3 SV=1
**tr|A0R5N2|A0R5N2_MYCS2**	MSMEG_6251	-3,573	6,449	Uncharacterized protein OS=Mycobacterium smegmatis (strain ATCC 700084 / mc(2)155) GN=MSMEG_6251 PE=4 SV=1
**tr|A0QPD6|A0QPD6_MYCS2**	MSMEG_0361	-3,565	3,44	Glycosyl hydrolase family protein 3 OS=Mycobacterium smegmatis (strain ATCC 700084 / mc(2)155) GN=MSMEG_0361 PE=1 SV=1
**tr|A0R4N8|A0R4N8_MYCS2**	MSMEG_5900	-3,52	4,271	Virulence factor Mce family protein OS=Mycobacterium smegmatis (strain ATCC 700084 / mc(2)155) GN=mce4A PE=4 SV=1
**tr|A0R084|A0R084_MYCS2**	MSMEG_4295	-3,503	4,068	Hydrolase, alpha/beta fold family protein OS=Mycobacterium smegmatis (strain ATCC 700084 / mc(2)155) GN=MSMEG_4295 PE=4 SV=1
**tr|A0R3B5|A0R3B5_MYCS2**	MSMEG_5412	-3,433	4,082	Immunogenic protein MPT63 OS=Mycobacterium smegmatis (strain ATCC 700084 / mc(2)155) GN=MSMEG_5412 PE=4 SV=1
**tr|A0R4N7|A0R4N7_MYCS2**	MSMEG_5899	-3,404	5,241	MCE-family protein MCE4b OS=Mycobacterium smegmatis (strain ATCC 700084 / mc(2)155) GN=mce4B PE=4 SV=1
**tr|A0QRR9|A0QRR9_MYCS2**	MSMEG_1215	-3,303	7,123	Serine/threonine-protein kinase PknE, putative OS=Mycobacterium smegmatis (strain ATCC 700084 / mc(2)155) GN=MSMEG_1215 PE=4 SV=1
**tr|A0QRN0|A0QRN0_MYCS2**	MSMEG_1176	-3,298	4,849	PE-PPE, C-terminal domain protein OS=Mycobacterium smegmatis (strain ATCC 700084 / mc(2)155) GN=MSMEG_1176 PE=4 SV=1
**tr|A0QXZ3|A0QXZ3_MYCS2**	MSMEG_3477	-3,263	2,482	Cell wall-associated hydrolases (Invasion-associated proteins) OS=Mycobacterium smegmatis (strain ATCC 700084 / mc(2)155) GN=MSMEG_3477 PE=4 SV=1
**tr|A0R4M5|A0R4M5_MYCS2**	MSMEG_5887	-3,075	2,306	Intersectin-EH binding protein Ibp1 OS=Mycobacterium smegmatis (strain ATCC 700084 / mc(2)155) GN=MSMEG_5887 PE=4 SV=1
**tr|A0QQP4|A0QQP4_MYCS2**	MSMEG_0828	-2,972	5,989	Immunogenic protein MPT63 OS=Mycobacterium smegmatis (strain ATCC 700084 / mc(2)155) GN=MSMEG_0828 PE=4 SV=1
**tr|A0R0E8|A0R0E8_MYCS2**	MSMEG_4360	-2,951	4,136	Putative secreted protein OS=Mycobacterium smegmatis (strain ATCC 700084 / mc(2)155) GN=MSMEG_4360 PE=4 SV=1
**tr|A0R3K2|A0R3K2_MYCS2**	MSMEG_5502	-2,943	3,893	AAA ATPase containing von Willebrand factor type A (VWA) domain-like protein OS=Mycobacterium smegmatis (strain ATCC 700084 / mc(2)155) GN=MSMEG_5502 PE=4 SV=1

**tr|A0QQ04|A0QQ04_MYCS2**	gabT	-2,934	2,271	4-aminobutyrate aminotransferase OS=Mycobacterium smegmatis (strain ATCC 700084 / mc(2)155) GN=gabT PE=1 SV=1
**sp|A0QQU5|CH601_MYCS2**	groL1	-2,896	3,603	60 kDa chaperonin 1 OS=Mycobacterium smegmatis (strain ATCC 700084 / mc(2)155) GN=groL1 PE=1 SV=1
**tr|A0R519|A0R519_MYCS2**	MSMEG_6033	-2,881	2,626	Uncharacterized protein OS=Mycobacterium smegmatis (strain ATCC 700084 / mc(2)155) GN=MSMEG_6033 PE=4 SV=1
**tr|A0QPF6|A0QPF6_MYCS2**	mmpl4a	-2,791	6,36	Mmp14a protein OS=Mycobacterium smegmatis (strain ATCC 700084 / mc(2)155) GN=mmpl4a PE=4 SV=1
**tr|A0R4D6|A0R4D6_MYCS2**	MSMEG_5796	-2,786	2,183	Glycine cleavage T protein (Aminomethyl transferase) OS=Mycobacterium smegmatis (strain ATCC 700084 / mc(2)155) GN=MSMEG_5796 PE=3 SV=1
**tr|A0R486|A0R486_MYCS2**	MSMEG_5741	-2,777	3,77	Uncharacterized protein OS=Mycobacterium smegmatis (strain ATCC 700084 / mc(2)155) GN=MSMEG_5741 PE=4 SV=1
**tr|A0R228|A0R228_MYCS2**	MSMEG_4965	-2,774	5,383	Uncharacterized protein OS=Mycobacterium smegmatis (strain ATCC 700084 / mc(2)155) GN=MSMEG_4965 PE=4 SV=1
**tr|A0R754|A0R754_MYCS2**	MSMEG_6784	-2,772	5,376	Beta-lactamase OS=Mycobacterium smegmatis (strain ATCC 700084 / mc(2)155) GN=MSMEG_6784 PE=4 SV=1
**tr|I7FXA5|I7FXA5_MYCS2**	MSMEI_0897	-2,766	4,561	Uncharacterized protein
**tr|A0R2Q4|A0R2Q4_MYCS2**	MSMEG_5196	-2,692	5,047	Beta-Ig-H3/fasciclin OS=Mycobacterium smegmatis (strain ATCC 700084 / mc(2)155) GN=MSMEG_5196 PE=4 SV=1
**tr|A0QXP7|A0QXP7_MYCS2**	MSMEG_3378	-2,688	3,236	Beta-lactamase OS=Mycobacterium smegmatis (strain ATCC 700084 / mc(2)155) GN=MSMEG_3378 PE=4 SV=1
**tr|A0QY05|A0QY05_MYCS2**	MSMEG_3489	-2,652	2,064	Uncharacterized protein OS=Mycobacterium smegmatis (strain ATCC 700084 / mc(2)155) GN=MSMEG_3489 PE=4 SV=1
**tr|A0QXB8|A0QXB8_MYCS2**	MSMEG_3244	-2,647	5,293	Uncharacterized protein OS=Mycobacterium smegmatis (strain ATCC 700084 / mc(2)155) GN=MSMEG_3244 PE=4 SV=1
**tr|A0QSW8|A0QSW8_MYCS2**	MSMEG_1629	-2,624	2,406	Uncharacterized protein OS=Mycobacterium smegmatis (strain ATCC 700084 / mc(2)155) GN=MSMEG_1629 PE=4 SV=1
**tr|A0R1E4|A0R1E4_MYCS2**	MSMEG_4722	-2,62	3,149	Short-chain dehydrogenase OS=Mycobacterium smegmatis (strain ATCC 700084 / mc(2)155) GN=MSMEG_4722 PE=4 SV=1
**tr|A0QP10|A0QP10_MYCS2**	MSMEG_0233	-2,601	2,761	Lipoprotein Lpps OS=Mycobacterium smegmatis (strain ATCC 700084 / mc(2)155) GN=MSMEG_0233 PE=4 SV=1

**tr|A0QS92|A0QS92_MYCS2**	MSMEG_1394	-2,581	2,076	Conserved transmembrane protein OS=Mycobacterium smegmatis (strain ATCC 700084 / mc(2)155) GN=MSMEG_1394 PE=4 SV=1
**tr|A0R330|A0R330_MYCS2**	MSMEG_5322	-2,577	4,067	Uncharacterized protein OS=Mycobacterium smegmatis (strain ATCC 700084 / mc(2)155) GN=MSMEG_5322 PE=4 SV=1
**sp|A0R6E3|Y6518_MYCS2**	MSMEG_6518	-2,57	6,374	Uncharacterized protein MSMEG_6518/MSMEI_6344 OS=Mycobacterium smegmatis (strain ATCC 700084 / mc(2)155) GN=MSMEG_6518 PE=1 SV=1
**sp|A0QVB9|EFTS_MYCS2**	tsf	-2,517	2,965	Elongation factor Ts OS=Mycobacterium smegmatis (strain ATCC 700084 / mc(2)155) GN=tsf PE=1 SV=1
**sp|A0R199|TIG_MYCS2**	tig	-2,506	2,516	Trigger factor OS=Mycobacterium smegmatis (strain ATCC 700084 / mc(2)155) GN=tig PE=1 SV=1
**sp|A0QSS4|CH602_MYCS2**	groL2	-2,501	3,518	60 kDa chaperonin 2 OS=Mycobacterium smegmatis (strain ATCC 700084 / mc(2)155) GN=groL2 PE=1 SV=1
**tr|A0QYD4|A0QYD4_MYCS2**	MSMEG_3619	-2,497	4,973	Short chain dehydrogenase OS=Mycobacterium smegmatis (strain ATCC 700084 / mc(2)155) GN=MSMEG_3619 PE=4 SV=1
**sp|A0QPU4|MSPB_MYCS2**	mspB	-2,459	3,388	Porin MspB OS=Mycobacterium smegmatis (strain ATCC 700084 / mc(2)155) GN=mspB PE=1 SV=1
**tr|A0R6C8|A0R6C8_MYCS2**	MSMEG_6504	-2,444	2,228	Uncharacterized protein OS=Mycobacterium smegmatis (strain ATCC 700084 / mc(2)155) GN=MSMEG_6504 PE=4 SV=1
**tr|A0QYH7|A0QYH7_MYCS2**	MSMEG_3662	-2,415	8,714	LysM domain protein OS=Mycobacterium smegmatis (strain ATCC 700084 / mc(2)155) GN=MSMEG_3662 PE=1 SV=1
**tr|A0R582|A0R582_MYCS2**	MSMEG_6099	-2,413	2,511	Probable conserved transmembrane protein rich in alanine OS=Mycobacterium smegmatis (strain ATCC 700084 / mc(2)155) GN=MSMEG_6099 PE=4 SV=1
**tr|A0QYR0|A0QYR0_MYCS2**	MSMEG_3751	-2,401	8,179	Cytotoxin/hemolysin, TlyA OS=Mycobacterium smegmatis (strain ATCC 700084 / mc(2)155) GN=MSMEG_3751 PE=4 SV=1
**sp|A0R561|CARD_MYCS2**	carD	-2,393	2,299	RNA polymerase-binding transcription factor CarD OS=Mycobacterium smegmatis (strain ATCC 700084 / mc(2)155) GN=carD PE=1 SV=1
**sp|A0QV10|Y2408_MYCS2**	MSMEG_2408	-2,38	2,555	Uncharacterized oxidoreductase MSMEG_2408/MSMEI_2347 OS=Mycobacterium smegmatis (strain ATCC 700084 / mc(2)155) GN=MSMEG_2408 PE=1 SV=1
**tr|A0R5U8|A0R5U8_MYCS2**	MSMEG_6319	-2,374	5,177	Penicillin-binding protein OS=Mycobacterium smegmatis (strain ATCC 700084 / mc(2)155) GN=MSMEG_6319 PE=4 SV=1
**sp|A0R006|WAG31_MYCS2**	wag31	-2,346	4,711	Cell wall synthesis protein Wag31 OS=Mycobacterium smegmatis (strain ATCC 700084 / mc(2)155) GN=wag31 PE=1 SV=1

**tr|A0QVQ7|A0QVQ7_MYCS2**	MSMEG_2658	-2,311	3,453	Beta-lactamase OS=Mycobacterium smegmatis (strain ATCC 700084 / mc(2)155) GN=MSMEG_2658 PE=3 SV=1
**tr|A0QTI1|A0QTI1_MYCS2**	selD	-2,278	2,866	Selenide, water dikinase OS=Mycobacterium smegmatis (strain ATCC 700084 / mc(2)155) GN=selD PE=3 SV=1
**tr|A0QPF7|A0QPF7_MYCS2**	mmpL4b	-2,265	10,918	Mmpl4b OS=Mycobacterium smegmatis (strain ATCC 700084 / mc(2)155) GN=mmpL4b PE=4 SV=1
**tr|A0R2U7|A0R2U7_MYCS2**	glpX	-2,263	2,859	Fructose-1,6-bisphosphatase OS=Mycobacterium smegmatis (strain ATCC 700084 / mc(2)155) GN=glpX PE=3 SV=1
**sp|O85501|NDK_MYCS2**	ndk	-2,261	2,902	Nucleoside diphosphate kinase OS=Mycobacterium smegmatis (strain ATCC 700084 / mc(2)155) GN=ndk PE=3 SV=1
**tr|A0QS24|A0QS24_MYCS2**	MSMEG_1322	-2,238	2,43	ErfK/YbiS/YcfS/YnhG family protein OS=Mycobacterium smegmatis (strain ATCC 700084 / mc(2)155) GN=MSMEG_1322 PE=4 SV=1
**tr|A0QXT5|A0QXT5_MYCS2**	MSMEG_3419	-2,234	2,263	Uncharacterized protein OS=Mycobacterium smegmatis (strain ATCC 700084 / mc(2)155) GN=MSMEG_3419 PE=4 SV=1
**tr|A0QNJ4|A0QNJ4_MYCS2**	MSMEG_0064	-2,215	3,13	PPE family protein OS=Mycobacterium smegmatis (strain ATCC 700084 / mc(2)155) GN=ppe68 PE=4 SV=1
**tr|A0QYD3|A0QYD3_MYCS2**	MSMEG_3618	-2,209	2,771	Alanine and proline-rich secreted protein apa OS=Mycobacterium smegmatis (strain ATCC 700084 / mc(2)155) GN=MSMEG_3618 PE=4 SV=1
**tr|A0QTF7|A0QTF7_MYCS2**	MSMEG_1824	-2,198	4,524	Transcriptional regulator, LytR family protein OS=Mycobacterium smegmatis (strain ATCC 700084 / mc(2)155) GN=MSMEG_1824 PE=4 SV=1
**tr|A0R7J1|A0R7J1_MYCS2**	MSMEG_6935	-2,187	4,953	N-acetylmuramoyl-L-alanine amidase OS=Mycobacterium smegmatis (strain ATCC 700084 / mc(2)155) GN=MSMEG_6935 PE=4 SV=1
**tr|A0QVC8|A0QVC8_MYCS2**	MSMEG_2529	-2,131	2,742	D-isomer specific 2-hydroxyacid dehydrogenase NAD-binding protein OS=Mycobacterium smegmatis (strain ATCC 700084 / mc(2)155) GN=MSMEG_2529 PE=3 SV=1
**tr|A0R2I9|A0R2I9_MYCS2**	MSMEG_5131	-2,121	3,578	Uncharacterized protein OS=Mycobacterium smegmatis (strain ATCC 700084 / mc(2)155) GN=MSMEG_5131 PE=4 SV=1
**tr|A0R716|A0R716_MYCS2**	MSMEG_6746	-2,078	2,041	Oxidoreductase, aldo/keto reductase family OS=Mycobacterium smegmatis (strain ATCC 700084 / mc(2)155) GN=MSMEG_6746 PE=4 SV=1
**tr|A0R239|A0R239_MYCS2**	MSMEG_4976	-2,073	2,534	Isochorismatase hydrolase OS=Mycobacterium smegmatis (strain ATCC 700084 / mc(2)155) GN=MSMEG_4976 PE=4 SV=1
**sp|A0QX87|HIS6_MYCS2**	hisF	-2,065	2,292	Imidazole glycerol phosphate synthase subunit HisF OS=Mycobacterium smegmatis (strain ATCC 700084 / mc(2)155) GN=hisF PE=3 SV=1

**tr|A0QRY7|A0QRY7_MYCS2**	MSMEG_1285	-2,057	4,819	SEC-C motif domain protein OS=Mycobacterium smegmatis (strain ATCC 700084 / mc(2)155) GN=MSMEG_1285 PE=4 SV=1
**sp|A0QQL0|THIG_MYCS2**	thiG	-2,053	2,807	Thiazole synthase OS=Mycobacterium smegmatis (strain ATCC 700084 / mc(2)155) GN=thiG PE=1 SV=1
**tr|A0R5R9|A0R5R9_MYCS2**	MSMEG_6289	-2,042	2,991	Trypsin OS=Mycobacterium smegmatis (strain ATCC 700084 / mc(2)155) GN=MSMEG_6289 PE=4 SV=1
**tr|A0QNR0|A0QNR0_MYCS2**	MSMEG_0132	-2,003	3,344	Conserved hypothetical integral membrane protein Yrbe1a OS=Mycobacterium smegmatis (strain ATCC 700084 / mc(2)155) GN=yrbE1A PE=4 SV=1
**tr|A0QY12|A0QY12_MYCS2**	MSMEG_3496	-2,003	3,638	MmpL4 protein OS=Mycobacterium smegmatis (strain ATCC 700084 / mc(2)155) GN=mmpL5 PE=4 SV=1
**tr|A0R5N6|A0R5N6_MYCS2**	MSMEG_6255	-2	5,84	Uncharacterized protein OS=Mycobacterium smegmatis (strain ATCC 700084 / mc(2)155) GN=MSMEG_6255 PE=4 SV=1
**tr|A0QPJ7|A0QPJ7_MYCS2**	MSMEG_0422	-1,979	2,063	PEP phosphonomutase OS=Mycobacterium smegmatis (strain ATCC 700084 / mc(2)155) GN=MSMEG_0422 PE=4 SV=1
**tr|A0R782|A0R782_MYCS2**	MSMEG_6815	-1,97	3,556	Secreted protein OS=Mycobacterium smegmatis (strain ATCC 700084 / mc(2)155) GN=MSMEG_6815 PE=4 SV=1
**tr|A0QQP1|A0QQP1_MYCS2**	thiD	-1,966	2,719	Phosphomethylpyrimidine kinase OS=Mycobacterium smegmatis (strain ATCC 700084 / mc(2)155) GN=thiD PE=4 SV=1
**tr|A0QP02|A0QP02_MYCS2**	MSMEG_0225	-1,945	4,577	MmpL4 protein OS=Mycobacterium smegmatis (strain ATCC 700084 / mc(2)155) GN=mmpL5 PE=4 SV=1
**tr|A0QS80|A0QS80_MYCS2**	MSMEG_1382	-1,934	6,525	MmpL5 protein OS=Mycobacterium smegmatis (strain ATCC 700084 / mc(2)155) GN=mmpL5 PE=4 SV=1
**tr|A0QU76|A0QU76_MYCS2**	MSMEG_2107	-1,929	4,696	Uncharacterized protein OS=Mycobacterium smegmatis (strain ATCC 700084 / mc(2)155) GN=MSMEG_2107 PE=4 SV=1
**tr|A0R5H3|A0R5H3_MYCS2**	MSMEG_6191	-1,917	6,812	Putative endoribonuclease L-PSP family OS=Mycobacterium smegmatis (strain ATCC 700084 / mc(2)155) GN=MSMEG_6191 PE=4 SV=1
**tr|A0QU56|A0QU56_MYCS2**	MSMEG_2083	-1,894	2,522	Inositol monophosphatase OS=Mycobacterium smegmatis (strain ATCC 700084 / mc(2)155) GN=MSMEG_2083 PE=4 SV=1
**tr|A0R4B6|A0R4B6_MYCS2**	MSMEG_5775	-1,894	4,006	LytR/CpsA/Psr family protein OS=Mycobacterium smegmatis (strain ATCC 700084 / mc(2)155) GN=MSMEG_5775 PE=4 SV=1
**tr|A0QZ55|A0QZ55_MYCS2**	MSMEG_3903	-1,888	2,646	Low molecular weight antigen CFP2 (Low molecular weight protein antigen 2) (CFP-2) OS=Mycobacterium smegmatis (strain ATCC 700084 / mc(2)155) GN=MSMEG_3903 PE=4 SV=1

**tr|A0QSM1|A0QSM1_MYCS2**	MSMEG_1526	-1,881	4,101	Cutinase OS=Mycobacterium smegmatis (strain ATCC 700084 / mc(2)155) GN=cut2 PE=3 SV=1
**tr|A0R3L1|A0R3L1_MYCS2**	MSMEG_5512	-1,867	2,468	Magnesium chelatase OS=Mycobacterium smegmatis (strain ATCC 700084 / mc(2)155) GN=MSMEG_5512 PE=4 SV=1
**tr|I7G1T1|I7G1T1_MYCS2**	mmpL	-1,866	7,148	MmpL
**tr|A0R4M2|A0R4M2_MYCS2**	MSMEG_5884	-1,858	5,112	3-hydroxyisobutyrate dehydrogenase family protein OS=Mycobacterium smegmatis (strain ATCC 700084 / mc(2)155) GN=MSMEG_5884 PE=4 SV=1
**tr|A0QX30|A0QX30_MYCS2**	MSMEG_3153	-1,833	2,316	Uncharacterized protein OS=Mycobacterium smegmatis (strain ATCC 700084 / mc(2)155) GN=MSMEG_3153 PE=4 SV=1
**tr|A0QRU8|A0QRU8_MYCS2**	MSMEG_1245	-1,83	2,371	Phosphoadenosine phosphosulfate reductase OS=Mycobacterium smegmatis (strain ATCC 700084 / mc(2)155) GN=MSMEG_1245 PE=4 SV=1
**tr|A0R529|A0R529_MYCS2**	otsB	-1,823	2,321	Trehalose 6-phosphate phosphatase OS=Mycobacterium smegmatis (strain ATCC 700084 / mc(2)155) GN=otsB PE=3 SV=1
**tr|I7G891|I7G891_MYCS2**	aroB	-1,821	5,045	3-dehydroquinate synthase OS=Mycobacterium smegmatis (strain ATCC 700084 / mc(2)155) GN=aroB PE=3 SV=1
**tr|A0QS41|A0QS41_MYCS2**	MSMEG_1341	-1,804	2,959	MaoC family protein OS=Mycobacterium smegmatis (strain ATCC 700084 / mc(2)155) GN=MSMEG_1341 PE=1 SV=1
**tr|A0R6G7|A0R6G7_MYCS2**	MSMEG_6542	-1,8	2,081	B12 binding domain protein OS=Mycobacterium smegmatis (strain ATCC 700084 / mc(2)155) GN=MSMEG_6542 PE=4 SV=1
**sp|A0R1X0|MURI_MYCS2**	murI	-1,798	4,333	Glutamate racemase OS=Mycobacterium smegmatis (strain ATCC 700084 / mc(2)155) GN=murI PE=1 SV=1
**tr|A0QTE3|A0QTE3_MYCS2**	MSMEG_1809	-1,779	3,103	Sulfurtransferase OS=Mycobacterium smegmatis (strain ATCC 700084 / mc(2)155) GN=sseA PE=4 SV=1
**tr|A0QTQ9|A0QTQ9_MYCS2**	MSMEG_1931	-1,775	6,82	Uncharacterized protein OS=Mycobacterium smegmatis (strain ATCC 700084 / mc(2)155) GN=MSMEG_1931 PE=4 SV=1
**tr|A0QNM4|A0QNM4_MYCS2**	MSMEG_0096	-1,772	2,735	Peroxisomal hydratase-dehydrogenase-epimerase OS=Mycobacterium smegmatis (strain ATCC 700084 / mc(2)155) GN=MSMEG_0096 PE=3 SV=1
**sp|Q59560|RECA_MYCS2**	recA	-1,729	2,072	Protein RecA OS=Mycobacterium smegmatis (strain ATCC 700084 / mc(2)155) GN=recA PE=1 SV=1
**tr|A0QTT5|A0QTT5_MYCS2**	MSMEG_1957	-1,715	3,248	Uncharacterized protein OS=Mycobacterium smegmatis (strain ATCC 700084 / mc(2)155) GN=MSMEG_1957 PE=4 SV=1

**tr|A0R656|A0R656_MYCS2**	MSMEG_6431	-1,703	4,501	Uncharacterized protein OS=Mycobacterium smegmatis (strain ATCC 700084 / mc(2)155) GN=MSMEG_6431 PE=4 SV=1
**tr|A0QQC2|A0QQC2_MYCS2**	MSMEG_0703	-1,694	3,582	Uncharacterized protein OS=Mycobacterium smegmatis (strain ATCC 700084 / mc(2)155) GN=MSMEG_0703 PE=4 SV=1
**tr|A0R4K9|A0R4K9_MYCS2**	MSMEG_5871	-1,694	4,444	HIT family protein OS=Mycobacterium smegmatis (strain ATCC 700084 / mc(2)155) GN=MSMEG_5871 PE=4 SV=1
**sp|A0QTP2|SIGH_MYCS2**	sigH	-1,69	2,107	ECF RNA polymerase sigma factor SigH OS=Mycobacterium smegmatis (strain ATCC 700084 / mc(2)155) GN=sigH PE=1 SV=2
**tr|A0QYD5|A0QYD5_MYCS2**	MSMEG_3620	-1,675	4,132	Putative luciferase-like oxidoreductase OS=Mycobacterium smegmatis (strain ATCC 700084 / mc(2)155) GN=MSMEG_3620 PE=4 SV=1
**tr|A0QYF5|A0QYF5_MYCS2**	glcB	-1,659	2,419	Malate synthase G OS=Mycobacterium smegmatis (strain ATCC 700084 / mc(2)155) GN=glcB PE=3 SV=1
**tr|A0QSX6|A0QSX6_MYCS2**	MSMEG_1637	-1,65	3,743	Histidine kinase OS=Mycobacterium smegmatis (strain ATCC 700084 / mc(2)155) GN=MSMEG_1637 PE=4 SV=1
**tr|I7G7R7|I7G7R7_MYCS2**	galU	-1,648	2,944	Putative UTP--glucose-1-phosphate uridylyltransferase OS=Mycobacterium smegmatis (strain ATCC 700084 / mc(2)155) GN=galU PE=4 SV=1
**tr|A0QP61|A0QP61_MYCS2**	MSMEG_0284	-1,647	2,216	NAD(P)H dehydrogenase (Quinone) OS=Mycobacterium smegmatis (strain ATCC 700084 / mc(2)155) GN=MSMEG_0284 PE=4 SV=1
**sp|A0R4C9|THTR_MYCS2**	MSMEG_5789	-1,646	2,276	Putative thiosulfate sulfurtransferase OS=Mycobacterium smegmatis (strain ATCC 700084 / mc(2)155) GN=MSMEG_5789 PE=1 SV=1
**sp|A0QSG7|RL30_MYCS2**	rpmD	-1,639	3,3	50S ribosomal protein L30 OS=Mycobacterium smegmatis (strain ATCC 700084 / mc(2)155) GN=rpmD PE=3 SV=1
**tr|A0QTE7|A0QTE7_MYCS2**	MSMEG_1813	-1,63	3,347	Propionyl-CoA carboxylase beta chain OS=Mycobacterium smegmatis (strain ATCC 700084 / mc(2)155) GN=accD5 PE=4 SV=1
**tr|I7GAS0|I7GAS0_MYCS2**	MSMEI_3793	-1,63	2,836	5'-3' exonuclease OS=Mycobacterium smegmatis (strain ATCC 700084 / mc(2)155) GN=MSMEI_3793 PE=4 SV=1
**tr|A0R2P1|A0R2P1_MYCS2**	MSMEG_5183	-1,614	2,378	3-Hydroxyacyl-CoA dehydrogenase OS=Mycobacterium smegmatis (strain ATCC 700084 / mc(2)155) GN=MSMEG_5183 PE=3 SV=1

Table 2: List of proteins of *Msm* significantly enriched in the PM fraction.

Proteins of *Msm* identified by quantitative proteomics analyses as specifically enriched in the PM fraction as compared to the MMCW fraction (ratio PM/MMCW > 3 and p-value < 0.01) are listed. The MS intensity ratios are expressed in Log_2 and the p-values are expressed in $-Log_{10}$.

Accession	Gene Name	Ratio	P-Value	Description
**tr|A0QVI4|A0QVI4_MYCS2**	MSMEG_2585	1,59	3,755	Uncharacterized protein OS=Mycobacterium smegmatis (strain ATCC 700084 / mc(2)155) GN=MSMEG_2585 PE=4 SV=1
**tr|A0QV22|A0QV22_MYCS2**	MSMEG_2420	1,599	3,281	Uncharacterized protein OS=Mycobacterium smegmatis (strain ATCC 700084 / mc(2)155) GN=MSMEG_2420 PE=4 SV=1
**tr|A0QYL7|A0QYL7_MYCS2**	MSMEG_3704	1,611	6,809	3-alpha-hydroxysteroid dehydrogenase OS=Mycobacterium smegmatis (strain ATCC 700084 / mc(2)155) GN=MSMEG_3704 PE=4 SV=1
**sp|A0QSD4|RL2_MYCS2**	rplB	1,621	5,289	50S ribosomal protein L2 OS=Mycobacterium smegmatis (strain ATCC 700084 / mc(2)155) GN=rplB PE=1 SV=1
**tr|A0QR72|A0QR72_MYCS2**	MSMEG_1009	1,627	7,737	Cytochrome P450 CYP136 OS=Mycobacterium smegmatis (strain ATCC 700084 / mc(2)155) GN=cyp136 PE=3 SV=1
**tr|A0QTV7|A0QTV7_MYCS2**	MSMEG_1981	1,632	3,727	Uncharacterized protein OS=Mycobacterium smegmatis (strain ATCC 700084 / mc(2)155) GN=MSMEG_1981 PE=4 SV=1
**tr|A0R054|A0R054_MYCS2**	MSMEG_4265	1,635	5,096	MmpS3 protein OS=Mycobacterium smegmatis (strain ATCC 700084 / mc(2)155) GN=mmpS3 PE=4 SV=1
**tr|A0QT33|A0QT33_MYCS2**	MSMEG_1695	1,649	2,163	Phosphoglucomutase/phosphomannomutase OS=Mycobacterium smegmatis (strain ATCC 700084 / mc(2)155) GN=pmmB PE=3 SV=1
**tr|A0R5I8|A0R5I8_MYCS2**	MSMEG_6207	1,691	2,358	Uncharacterized protein OS=Mycobacterium smegmatis (strain ATCC 700084 / mc(2)155) GN=MSMEG_6207 PE=4 SV=1
**tr|A0QS52|A0QS52_MYCS2**	MSMEG_1353	1,7	5,189	ABC1 family protein OS=Mycobacterium smegmatis (strain ATCC 700084 / mc(2)155) GN=MSMEG_1353 PE=4 SV=1
**tr|A0QVB5|A0QVB5_MYCS2**	MSMEG_2517	1,715	2,189	Alpha/beta hydrolase fold protein OS=Mycobacterium smegmatis (strain ATCC 700084 / mc(2)155) GN=bphD PE=4 SV=1

**sp|A0R628|GLFT2_MYCS2**	glfT2	1,741	5,58	Galactofuranosyl transferase GlfT2 OS=Mycobacterium smegmatis (strain ATCC 700084 / mc(2)155) GN=glfT2 PE=3 SV=1
**tr|A0QXC8|A0QXC8_MYCS2**	MSMEG_3255	1,767	2,54	DoxX OS=Mycobacterium smegmatis (strain ATCC 700084 / mc(2)155) GN=MSMEG_3255 PE=4 SV=1
**tr|A0QQA3|A0QQA3_MYCS2**	MSMEG_0683	1,774	2,442	Uncharacterized protein OS=Mycobacterium smegmatis (strain ATCC 700084 / mc(2)155) GN=MSMEG_0683 PE=4 SV=1
**tr|A0QTP8|A0QTP8_MYCS2**	MSMEG_1920	1,787	4,261	Diacylglycerol kinase catalytic region OS=Mycobacterium smegmatis (strain ATCC 700084 / mc(2)155) GN=MSMEG_1920 PE=4 SV=1
**tr|A0QX34|A0QX34_MYCS2**	MSMEG_3157	1,787	4,469	Integral membrane protein OS=Mycobacterium smegmatis (strain ATCC 700084 / mc(2)155) GN=MSMEG_3157 PE=4 SV=1
**tr|A0R1C6|A0R1C6_MYCS2**	MSMEG_4703	1,788	7,682	Glycerol-3-phosphate acyltransferase OS=Mycobacterium smegmatis (strain ATCC 700084 / mc(2)155) GN=MSMEG_4703 PE=3 SV=1
**tr|A0QSW4|A0QSW4_MYCS2**	MSMEG_1625	1,818	3,526	Amino acid permease-associated OS=Mycobacterium smegmatis (strain ATCC 700084 / mc(2)155) GN=MSMEG_1625 PE=4 SV=1
**sp|A0R4H4|Y5835_MYCS2**	MSMEG_5835	1,827	6,707	KsdD-like steroid dehydrogenase MSMEG_5835 OS=Mycobacterium smegmatis (strain ATCC 700084 / mc(2)155) GN=MSMEG_5835 PE=1 SV=1
**sp|A0QNF5|CWSA_MYCS2**	cwsA	1,828	3,507	Cell wall synthesis protein CwsA OS=Mycobacterium smegmatis (strain ATCC 700084 / mc(2)155) GN=cwsA PE=1 SV=1
**tr|I7F768|I7F768_MYCS2**	MSMEI_0925	1,849	4,851	HAD-superfamily subfamily IB hydrolase, TIGR01490 OS=Mycobacterium smegmatis (strain ATCC 700084 / mc(2)155) GN=MSMEI_0925 PE=4 SV=1
**tr|A0QQ56|A0QQ56_MYCS2**	MSMEG_0633	1,861	4,847	PAP2 superfamily protein OS=Mycobacterium smegmatis (strain ATCC 700084 / mc(2)155) GN=MSMEG_0633 PE=4 SV=1
**tr|A0QPE8|A0QPE8_MYCS2**	MSMEG_0373	1,871	6,841	3-ketoacyl-CoA thiolase OS=Mycobacterium smegmatis (strain ATCC 700084 / mc(2)155) GN=fadA2 PE=3 SV=1
**tr|A0QUV1|A0QUV1_MYCS2**	MSMEG_2344	1,877	6,109	Dehydrogenase OS=Mycobacterium smegmatis (strain ATCC 700084 / mc(2)155) GN=MSMEG_2344 PE=4 SV=1
**tr|A0R5R4|A0R5R4_MYCS2**	MSMEG_6283	1,916	7,301	FAD binding domain protein OS=Mycobacterium smegmatis (strain ATCC 700084 / mc(2)155) GN=MSMEG_6283 PE=4 SV=1
**sp|A0QQS6|PSD_MYCS2**	psd	1,95	3,602	Phosphatidylserine decarboxylase proenzyme OS=Mycobacterium smegmatis (strain ATCC 700084 / mc(2)155) GN=psd PE=3 SV=1
**tr|A0R427|A0R427_MYCS2**	MSMEG_5682	1,955	4,933	Uncharacterized protein OS=Mycobacterium smegmatis (strain ATCC 700084 / mc(2)155) GN=MSMEG_5682 PE=4 SV=1

**tr|A0R041|A0R041_MYCS2**	MSMEG_4252	1,98	2,574	Uncharacterized protein OS=Mycobacterium smegmatis (strain ATCC 700084 / mc(2)155) GN=MSMEG_4252 PE=4 SV=1
**sp|A0QSG2|RS14Z_MYCS2**	rpsZ	1,997	2,192	30S ribosomal protein S14 type Z OS=Mycobacterium smegmatis (strain ATCC 700084 / mc(2)155) GN=rpsZ PE=3 SV=1
**sp|A0QSE0|RS17_MYCS2**	rpsQ	2,098	3,516	30S ribosomal protein S17 OS=Mycobacterium smegmatis (strain ATCC 700084 / mc(2)155) GN=rpsQ PE=1 SV=1
**tr|A0R555|A0R555_MYCS2**	MSMEG_6071	2,133	4,803	Conserved hypothetical Zn-dependent hydrolase OS=Mycobacterium smegmatis (strain ATCC 700084 / mc(2)155) GN=MSMEG_6071 PE=4 SV=1
**tr|A0R5R3|A0R5R3_MYCS2**	MSMEG_6282	2,148	3,578	KanY protein OS=Mycobacterium smegmatis (strain ATCC 700084 / mc(2)155) GN=MSMEG_6282 PE=4 SV=1
**tr|A0R2B0|A0R2B0_MYCS2**	MSMEG_5048	2,149	3,334	Putative membrane protein OS=Mycobacterium smegmatis (strain ATCC 700084 / mc(2)155) GN=MSMEG_5048 PE=4 SV=1
**tr|A0QUY1|A0QUY1_MYCS2**	MSMEG_2377	2,159	4,045	P49 protein OS=Mycobacterium smegmatis (strain ATCC 700084 / mc(2)155) GN=MSMEG_2377 PE=4 SV=1
**sp|A0QSD5|RS19_MYCS2**	rpsS	2,287	2,445	30S ribosomal protein S19 OS=Mycobacterium smegmatis (strain ATCC 700084 / mc(2)155) GN=rpsS PE=3 SV=1
**tr|A0QVU2|A0QVU2_MYCS2**	MSMEG_2695	2,346	2,577	35 kDa protein OS=Mycobacterium smegmatis (strain ATCC 700084 / mc(2)155) GN=MSMEG_2695 PE=4 SV=1
**tr|I7GDQ2|I7GDQ2_MYCS2**	MSMEI_5077	2,356	2,857	Uncharacterized protein OS=Mycobacterium smegmatis (strain ATCC 700084 / mc(2)155) GN=MSMEI_5077 PE=4 SV=1
**tr|A0R659|A0R659_MYCS2**	MSMEG_6434	2,363	4,03	Uncharacterized protein OS=Mycobacterium smegmatis (strain ATCC 700084 / mc(2)155) GN=MSMEG_6434 PE=4 SV=1
**sp|A0R043|PIMB_MYCS2**	pimB	2,402	7,174	GDP-mannose-dependent alpha-(1-6)-phosphatidylinositol monomannoside mannosyltransferase OS=Mycobacterium smegmatis (strain ATCC 700084 / mc(2)155) GN=pimB PE=1 SV=1
**tr|A0QR34|A0QR34_MYCS2**	MSMEG_0970	2,412	7,017	Phosphoglycerate mutase OS=Mycobacterium smegmatis (strain ATCC 700084 / mc(2)155) GN=MSMEG_0970 PE=4 SV=1
**tr|A0QXT4|A0QXT4_MYCS2**	MSMEG_3418	2,586	3,274	Uncharacterized protein OS=Mycobacterium smegmatis (strain ATCC 700084 / mc(2)155) GN=MSMEG_3418 PE=4 SV=1
**tr|A0QUV3|A0QUV3_MYCS2**	MSMEG_2347	2,748	6,669	Phytoene dehydrogenase OS=Mycobacterium smegmatis (strain ATCC 700084 / mc(2)155) GN=MSMEG_2347 PE=3 SV=1
**tr|A0R0W5|A0R0W5_MYCS2**	cysW	2,859	3,218	Sulfate ABC transporter, permease protein CysW OS=Mycobacterium smegmatis (strain ATCC 700084 / mc(2)155) GN=cysW PE=4 SV=1

**tr|A0QT47|A0QT47_MYCS2**	MSMEG_1709	2,872	9,318	Inner membrane ABC transporter permease protein YjfF OS=Mycobacterium smegmatis (strain ATCC 700084 / mc(2)155) GN=MSMEG_1709 PE=3 SV=1
**sp|A0QYU7|RL35_MYCS2**	rpmI	3,024	2,547	50S ribosomal protein L35 OS=Mycobacterium smegmatis (strain ATCC 700084 / mc(2)155) GN=rpmI PE=3 SV=1
**tr|A0R0X2|A0R0X2_MYCS2**	MSMEG_4538	3,568	2,216	Cysteine desulfurase OS=Mycobacterium smegmatis (strain ATCC 700084 / mc(2)155) GN=MSMEG_4538 PE=3 SV=1
**tr|A0R0X1|A0R0X1_MYCS2**	MSMEG_4537	5,639	2,005	Major membrane protein I OS=Mycobacterium smegmatis (strain ATCC 700084 / mc(2)155) GN=MSMEG_4537 PE=4 SV=1

Figure 1 : Lipoglycans analysis of *Mau* fractions.

Membranes of *Mau* were isolated and the lipoglycan content of each fraction, plasma membrane (PM) and mycomembrane-containing cell wals (MMCW) were analyzed on SDS-PAGE and Silver-stained after a trypsin treatment of the samples. **A**: Lipoglycan content of PM and MMCW of *Mau* harvested in the logarithmic phase of growth. **B**: Lipoglycan content of PM and MMCW of *Mau* harvested in the stationary phase of growth. The ratios of LAM and LM for each fraction were calculated using ImageLab. For logarithmic phase (**A**) versus stationary phase (**B**), the ratios of lipoarabinomannans (LAM) in PM and MMCW were, respectively, 3.4 and 2.4. Those of lipomannans (LM) in PM and MMCW were, respectively, 2.2 and 1.8. MW : molecular weight in kDa; PIM: phosphatidylinositolmannosides.

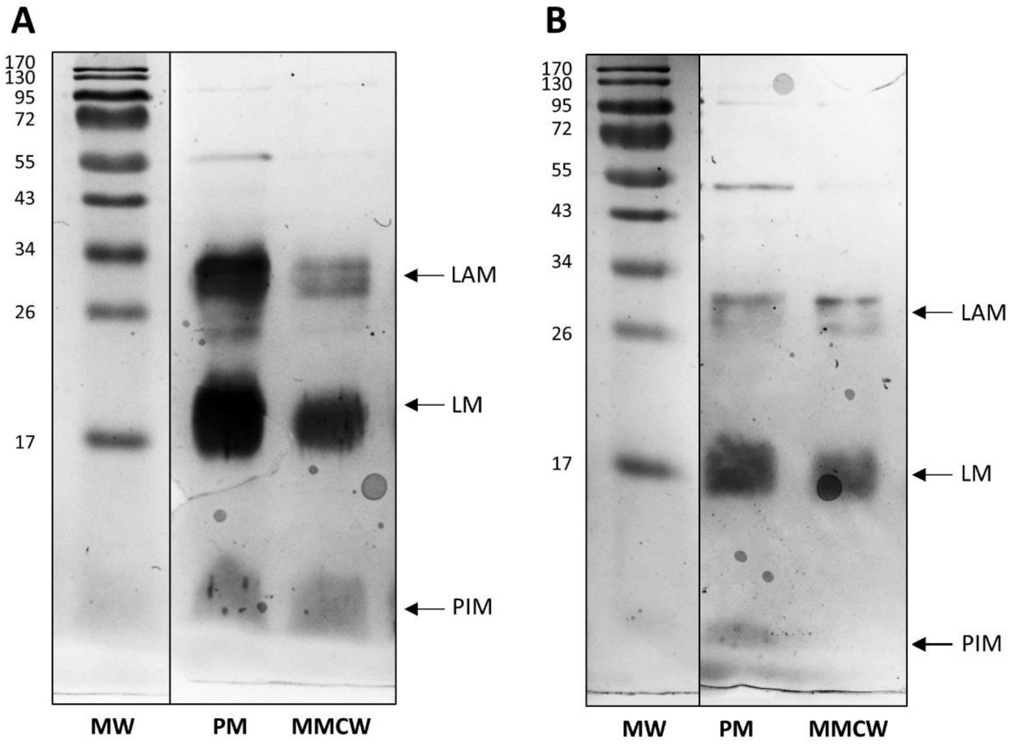

Supplement: Supplementary file 1 — Supplementary Information [file 41598_2017_12718_MOESM1_ESM.pdf]
